# Magnet design for a low-emittance storage ring

**DOI:** 10.1107/S160057751401666X

**Published:** 2014-08-27

**Authors:** Martin Johansson, Bengt Anderberg, Lars-Johan Lindgren

**Affiliations:** a MAX IV Laboratory, Lund University, Lund, Sweden; b AMACC AB, Uppsala, Sweden

**Keywords:** accelerator, magnet, magnet block, multi-bend achromat, low emittance

## Abstract

The MAX IV 3 GeV storage ring, currently under construction, pursues the goal of low electron beam emittance by using a multi-bend achromat magnet lattice, which is realised by having several consecutive magnet elements precision-machined out of a common solid iron block, 2.3–3.4 m long. With this magnet design solution, instead of having 1320 individual magnets, the MAX IV 3 GeV storage ring is built up using 140 integrated ‘magnet block’ units, containing all these magnet elements. Major features of this magnet block design are compactness, vibration stability and that the alignment of magnet elements within each unit is given by the mechanical accuracy of the CNC machining rather than individual field measurement and adjustment. This article presents practical engineering details of implementing this magnet design solution, and mechanical + magnetic field measurement results from the magnet production series. At the time of writing (spring 2014), the production series, which is totally outsourced to industry, is roughly half way through, with mechanical/magnetic QA conforming to specifications. It is the conclusion of the authors that the MAX IV magnet block concept, which has sometimes been described as new or innovative, is from a manufacturing point of view simply a collection of known mature production methods and measurement procedures, which can be executed at fixed cost with a low level of risk.

## Abstract 2

The magnet design of the MAX IV 3 GeV storage ring replaces the conventional support girder + discrete magnets scheme of previous third-generation light sources with a compact integrated design having several consecutive magnet elements precision-machined out of a common solid iron block.

## 1. Introduction   

**Figure 1:**
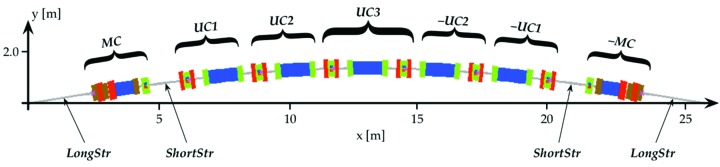
Schematic of one achromat of the MAX IV 3 GeV storage ring.

**Figure 2:**
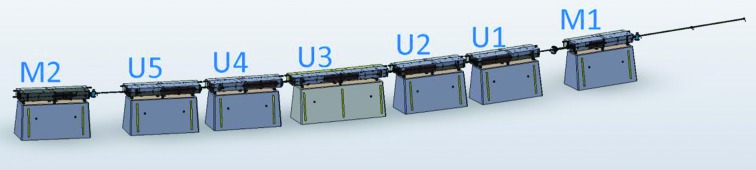
3D CAD assembly of magnets blocks, concrete supports and vacuum chamber of one achromat of the MAX IV 3 GeV storage ring (*cf.* Fig. 1 ▶).

The MAX IV synchrotron radiation facility, currently under construction/commissioning, will consist of a 528 m-circumference 20-achromat 3 GeV storage ring, a 96 m-circumference 12-achromat 1.5 GeV storage ring, and a full energy linac injector/SPF/FEL driver (Eriksson *et al.*, 2011 ▶). For the 3 GeV storage ring, a multi-bend achromat magnet lattice was chosen in order to minimize the electron beam emittance. A schematic of one achromat is shown in Fig. 1 ▶. The 3 GeV ring magnet design has each lattice cell (MC, UC1, UC2,…) realised as one integrated unit having several consecutive magnet elements precision-machined out of a common solid iron block, 2.3–3.4 m long, which we call a ‘magnet block’ (see Figs. 2 ▶ and 3 ▶). The key aspects in which the MAX IV 3 GeV ring magnet design stand out from previous third-generation synchrotron light sources is the magnet block concept, and the relatively small magnet aperture of Ø25 mm.

The small magnet aperture comes from the need to make the lattice as compact as possible, to maximize the length and number of lattice straight sections available for insertion devices for user beamlines. In the MAX IV 3 GeV storage ring design, the key enabling factor for compactness is the choice of NEG-coated copper vacuum chambers throughout the achromats. This linear pumping solution avoids the problem of a low vacuum conductance given by the small vacuum chamber aperture and reduces the space needed for lumped absorbers and pumps. This allowed choosing a magnet aperture of Ø25 mm, which has a direct impact on lattice compactness since the minimum distance between consecutive magnet elements is proportional to the magnet aperture. It should also be noted that smaller magnet aperture allows for higher field gradients, allowing shorter focusing magnet elements. There is also an indirect impact being that with smaller aperture the magnet excitation currents go down, decreasing the magnet coil cross sections, which makes it easier to pack the magnets close together.

The choice of the magnet block concept comes from considering several different factors:

(i) One aspect that has a direct impact on user performance is vibration stability, since vibration of focusing magnet elements translates to jitter of the stored electron beam position. The key factor for the eigenfrequency spectrum is the stiffness/mass ratio of the combined magnets + adjustment mechanisms + floor supports, which should be as high as possible. Compared with the conventional scheme of having several consecutive magnets mounted on a common girder, the MAX IV magnet block design decreases the moving mass and reduces the vibration amplitude by omitting the girder and placing the adjustment mechanisms directly under the magnets, on concrete supports (see Fig. 2 ▶). In FEM simulations, this results in the system eigenfrequencies being pushed up to ∼100 Hz for the combined concrete supports + adjustment mechanisms + magnet blocks. Since the vibrational power density spectrum is strongly decreasing with increasing frequency, the integrated vibrational displacement is thus radically decreased.

(ii) The second major reason for choosing the magnet block concept is that internal alignment between consecutive magnet elements is given by the machining tolerances within each yoke block, thus omitting the need of elaborate magnetic centres measurements and adjustments of the individual magnets on the girders.

(iii) The third factor is the reduction of the installation work. Instead of handling 1320 individual magnets, 140 magnet block units are installed, with cabling and plumbing already carried out before delivery.

Details of this magnet design, and results from the production series, are presented in the following subsections.

## 2. Lattice iteration and other basic specifications   

The MAX IV 3 GeV storage ring lattice consists of 20 identical multi-bend achromats, each consisting of two matching cells and five unit cells, with a ring circumference of 528 m and bare lattice emittance of 0.33 nm rad (Leemann *et al.*, 2009 ▶). The different magnet families in the 3 GeV ring lattice are listed in Tables 1–4. The detailed lattice and magnet design was iterated by setting up *Radia* 3D models of the dipole magnets, and representing the simulated magnetic field distribution as 12 consecutive slices per dipole in the lattice model. The minimum distance between consecutive magnet elements was taken to be equal to the magnet aperture and the maximum pole tip fields were chosen to be ∼0.5 T, assuming a magnet aperture of Ø25 mm. The resulting Detailed Design Report (DDR) versions of the lattice and magnets (Leemann, 2010*a* ▶; Lindgren & Anderberg, 2010 ▶) were used as a starting point for the detailed mechanical design (manufacturing drawings) of the magnet blocks. Detailed mechanical design and prototype magnet results introduced only minor updates (see Appendix *A* [App appa]). The 3 GeV ring lattice is presented in Appendix *A* [App appa], Table 10. Other major specifications that went into the magnet design are listed below:

(i) Each magnet block corresponds to a lattice cell.

(ii) Magnet aperture = Ø25 mm.

(iii) Design magnet cross sections around required space for photon beam pipe.

(iv) Coil cooling circuits pressure drop = 2 bar.

(v) Coil cooling water maximum temperature rise = 10 K.

(vi) Magnet families are series connected to the same power supplies^1^, individuals to be adjusted to equal strength by passively shunting part of the excitation current based on magnetic field measurement results.

The mechanical tolerances on the yoke-bottom/yoke-top blocks and other yoke parts were set as tight as assumed to be possible (see §7), with no adjustment possibility between the parts, meaning that the non-systematic field error level, and the alignment accuracy of the different magnets within each block, is as follows from those tolerances directly on assembly.

## 3. Magnet types   

For the different MAX IV 3 GeV storage-ring magnet types, the magnetic design in terms of pole profiles, return yoke cross sections and coil cross sections was presented as 2D/3D models in the MAX IV DDR (Lindgren & Anderberg, 2010 ▶), from which the magnet block drawings were made. It can be noted that no 3D field simulations of full magnet blocks were performed before generating the manufacturing drawings. The integrated yoke bottom and top blocks were drafted by simply taking the width and height of the magnet 2D model with the largest cross section, the dipole, and extending it over the length of a lattice cell; this under the assumption that by having return yoke field strengths below full saturation in each 2D design, the return flux of each magnet element will be kept mostly in its own portion of the common return yoke, and any smaller fraction of the return flux that spreads out over the adjacent magnet elements will go mostly through their return yokes, rather than taking the higher reluctance path through their pole gaps.

The following subsections present the different 3 GeV ring magnet types as built. The magnet cross sections are shown in the form of 2D field simulations^2^ created by exporting cross sections from the production 3D CAD models (Johansson, 2013 ▶). The 2D model geometries correspond to the top halves of the magnets, utilizing mid-plane symmetry, since the bottom and top magnet block halves are mirror identical.

### 3.1. Dipoles   

The MAX IV 3 GeV storage-ring dipoles are gradient dipoles, having a pole profile that produces a horizontally defocusing field gradient. The dipoles have pole face strips, providing a ±4% tuning range of the gradient. The placement of the dipoles is shown in Fig. 4 ▶ and the types are listed in Table 1 ▶.

Photographs of the two dipole types are shown in Fig. 5 ▶ and cross sections are shown in Fig. 6 ▶. The unit cell (DIP) and matching cell dipole (DIPm) have the same pole profile, but the matching cell dipole is shorter and has a soft end section with reduced field strength facing the long straights, which reduces the thermal load from synchrotron radiation in the downstream insertion device. The pole gap is 28 mm at *x* = 0, but, with the pole face strips taking 1.5 mm per pole, the available aperture is 25 mm. The pole is machined directly out of the bottom/top yoke blocks. In the longitudinal direction, the pole shape is curved, following a 3° arc for DIP and a 1.5° arc for DIPm, with larger radius through the soft end portion. The soft end is a loose pole piece with an extra air gap beneath. Both DIP and DIPm have field clamps at the entrance and exit sides, which are assembled into machined slots in the yoke bottom/top blocks. The field clamps reduce the sensitivity to coil shape variations making the field distribution more identical between magnets, and reduces fringe field overlap with the adjacent magnet elements. The pole face strips are copper plates which follow the same curvature as the poles. Their sideways profile has steps to give the correct field distribution. Over the poles, the pole face strips are dressed in kapton tape for electrical insulation and their sideways and vertical position is defined and kept in place by epoxy glass laminate holders and distance pieces.

### 3.2. Quadrupoles   

The MAX IV 3 GeV storage-ring quadrupoles are pure quadrupoles. Early drafts of the lattice had combined function quadrupole/sextupole magnets but this evolved into having two separate quadrupole magnets with a discrete sextupole in between in each unit cell. The placement of the quadrupoles is shown in Fig. 7 ▶ and the types are listed in Table 2 ▶.

The four different quadrupole families were designed with the same pole profile, and the same mechanical pole length for QFend and QDend, 231 mm, and the same length for QFm and QF, 131 mm, to be operated at different excitation currents (Lindgren & Anderberg, 2010 ▶). In the manufacturing drawings the outer sides of the pole tip were adjusted parallel to simplify the geometry, but initial field measurements showed that this restricted the iron cross section too much creating saturation in the pole root for QF. Therefore the pole tip design was adjusted for QFm and QF, leading to one design for QFend/QDend in the matching cell magnet blocks and one design for QFm/QF in the unit cell magnet blocks. The two quadrupole types are shown in Figs. 8 ▶ and 9 ▶. Both have the pole root machined directly out of the bottom/top yoke blocks, with the pole tip being a separate piece bolted in place over the coil. The pole tip vertical location is defined by the pole tip/pole root mating surface, and the sideways location is defined by a guiding slot in the pole root.

### 3.3. Sextupoles   

The placement of the MAX IV 3 GeV storage ring sextupoles is shown in Fig. 10 ▶ and the types are listed in Table 3 ▶.

For the five different sextupole families two different basic sextupole designs were made, one with 10 mm pole width and one with 20 mm pole width, both with 95 mm mechanical pole length. Integration with other subsystems led to there being two variants of each, giving one design for SDend, one for SFm, one for SD, and a common design for SFo/SFi to be operated at different excitation currents. Example photographs are shown in Fig. 11 ▶ and the cross sections of the four different variants are shown in Fig. 12 ▶. The sextupoles are designed as a standalone magnet with its own return yoke. The pole pieces are dismountable to allow installation of the coils. The location of each sextupole half is defined in the vertical direction by the mating surface to the large yoke bottom/top block, and sideways by a guiding slot in the bottom/top block (see Fig. 8 ▶). All sextupole magnets have a trim winding on each pole, which are wired individually to terminals on the inner side of the magnet blocks. The storage ring installation will have one trim power supply for each sextupole, with a relay card between, allowing remote switching between five different connection modes: normal quadrupole, skew quadrupole, auxiliary sextupole, horizontal corrector and vertical corrector.

### 3.4. Octupoles   

The MAX IV 3 GeV storage-ring octupoles are located in the matching cells, as shown in Fig. 13 ▶. The different types are listed in Table 4 ▶.

The octupoles are specified to have a tuning range of up to +100% of the lattice strength [see §2.3.3 of DDR (Leemann, 2010*a* ▶)], so the magnets are designed for twice the strength stated in Table 4 ▶. OXX and OXY are identical and operated at different excitation currents, but OYY has a larger aperture of Ø36 mm to allow passage of the photon beam pipe in matching cell 1. Both types have the same mechanical pole length, 95 mm, and the same coils and return yoke. Photographs and cross sections are shown in Figs. 14 ▶ and 15 ▶. Like the sextupoles, the octupoles are designed as stand­alone magnets split at the midplane with each half located by mating surfaces/guiding slots in the yoke bottom/top blocks. All octupoles have trim windings on each pole which, like for the sextupoles, can be remote switched between different connection modes: normal quadrupole, skew quadrupole, horizontal corrector and vertical corrector.

### 3.5. Correctors   

The placement of the MAX IV 3 GeV storage-ring slow orbit correctors is shown in Fig. 16 ▶. The specified steering angle per corrector is ±0.25 mrad.

The DDR had the correctors designed as pairs of window frame magnets with laminated yokes to allow fast steering (Lindgren & Anderberg, 2010 ▶). After it was seen that this design could not produce the required steering angle within the space available in the lattice (the space restriction was the distance between the BPM and OXX in the matching cells), a new design was made providing stronger field. This design required a more complex yoke shape, so the yoke material was changed from laminated steel to ferrite. This was the design version which was present in the call-for-tender version of the magnet blocks (Anderberg *et al.*, 2011 ▶). During contract negotiations (see §7) this design was changed from ferrite to iron in order to reduce complexity, meaning that fast steering will be done by separate magnets not integrated in the magnet blocks. The final design is shown in Fig. 17 ▶. The pole roots are machined directly out of the bottom/top yoke blocks, with the pole tips being separate pieces bolted in place over the coils. The choice of this magnet geometry with vertically oriented pole roots for the vertical corrector comes from the requirement of allowing passage of the photon beam pipe outside the magnet in unit cell 1.

## 4. Mechanical design   

As described above, a MAX IV 3 GeV storage-ring magnet block consists of a yoke bottom and a yoke top half assembled together with other yoke parts, coils, *etc*. The coils and parts in the bottom half are mounted on the yoke bottom, and the parts in the top half are mounted on the yoke top, meaning that the top half is easily dismounted from the bottom half to allow installation of the vacuum chamber. For each magnet block there is an assembly drawing with a bill of materials listing all included parts and sub-assemblies. An example assembly drawing is shown in Fig. S1 of the supporting information^3^, and the different magnet block types are listed in Table 5 ▶. Each magnet block type corresponds to one lattice cell, so which magnet elements are present in each block follows from the description in §3 above (and is written out explicitly in Appendix *A*, Table 10). The U1 and U2 magnet block types are identical except for the SFm/SFo sextupole, and the U5 and U4 magnet blocks are mirror identical to U1 and U2. M2 is mirror identical to M1 except for the correctors between the SDend and OYY magnet elements. The U3 layout is like U1/U2 or U4/U5 but symmetric around the dipole centre.

The major mechanical components of the MAX IV 3 GeV ring magnets are the yoke bottom and yoke top blocks, each of which are machined out of one solid iron block. Following the lattice layout, there are three basic types of yoke blocks, Mc1, Uc1–2 and Uc3. Each of these come in two variants, called ‘yoke bottom’ and ‘yoke top’, which are mirror identical around the horizontal midplane, except for that the yoke bottoms have attachment points to the support stand underneath, and each yoke top has five Ø30 mm conical holes on top serving as fiducial target seats. Then there are Uc4–5 yoke blocks which are mirror versions of Uc1–2, and Mc2 yoke blocks which are mirror identical to Mc1.^4^ The different types in are listed in Table 6 ▶.

Since the MAX IV magnet design depends on machining accuracy to align the magnets within each block, a key aspect of the design is how the yoke mechanical dimensions and tolerances are defined. The MAX IV solution is to have reference surfaces on each yoke bottom and yoke top block for defining all dimensions within the blocks, to use the same reference surfaces to align the magnet block top half to the bottom half, and to define the fiducial locations relative to the same reference surfaces. An example yoke block manufacturing drawing is shown in Fig. S2 of the supporting information, with these reference surfaces highlighted. Together the reference planes define a Cartesian coordinate system in which all the dimensions within the yoke block are given. The horizontal plane is defined as the large mating surface between the two yoke blocks (D in Fig. S2). The sideways reference surfaces are located at the outer side of the block near each end (A, B in Fig. S2); together they define the longitudinal direction. The longitudinal reference plane is located at the end of the block, defining the coordinate system origin (C in Fig. S2).

The yoke bottom and yoke top drawings listed in Table 6 ▶ contain all the dimensions needed to manufacture the blocks. On the drawing sheets a distinction is made between function critical dimensions, which have tolerances explicitly written out, and other surfaces for which a general tolerance class stated in the drawing frame applies. The critical surfaces can be sorted into the following categories,

(i) Vertical guiding surfaces: mating planes for quad pole tips, corrector pole tips, sextupole/octupole halves and dipole field clamps. Distance to the midplane is given with a tolerance of ±0.02 mm.

(ii) Sideways guiding surfaces: guiding slots for quad pole tips, corrector pole tips, sextupole/octupole halves and dipole field clamps. Distance to the sideways reference planes is given with a tolerance of ±0.02 mm on one side of the slot, and slot width is given with a −0+0.01 tolerance.

(iii) Dipole profile. The pole profile cross section is given as *x*,*y*-coordinates relative to a simplified beam trajectory^5^ with a surface shape tolerance of 0.04 mm (±0.02 mm) applying to the whole pole area, defined relative to D, A–B and C.^6^

(iv) The reference surfaces themselves: the midplane has a flatness tolerance of 0.04 mm, and the sideways/longitudinal reference surfaces have 0.02 mm perpendicularity relative to the midplane.

The longitudinal guiding features and pole ends also have explicit tolerances, but at a slightly relaxed level since they are less critical.

The part drawings of the loose yoke pieces are adapted to the yoke block drawing concept in that their dimensions are defined in the vertical direction relative to the mating surface to the large yoke block, and in the sideways direction relative to the key that goes in the guiding slot on the yoke blocks. The quadrupole pole tips and sextupole/octupole yoke halves have surface shape tolerance of 0.02 mm (±0.01 mm) and 0.04 mm (±0.02 mm) applying to the pole surfaces, defined relative to these reference surfaces. Some example part manufacturing drawings are shown in Fig. S3 of the supporting information.

As mentioned above, the yoke block reference surfaces are also used to define the location of the magnet block top half relative to the bottom half in assembly. In the vertical direction this is obvious, in that the mating surface between the two yoke block halves is the reference plane. Sideways and longitudinally the block halves are aligned relative to each other by guide blocks mounted directly on the reference surfaces (two of these can be seen in Fig. 11 ▶). We prefer this scheme over vertically oriented pins hidden inside the assembly or horizontally oriented pins drilled from the side in that the coordinate system definition in 3D mechanical measurement is straightforward and robust, that there is little risk of damaging the guiding features during handling, and that the alignment status between top half and bottom half can easily be checked by hand-held measurement tools in the assembled state.

## 5. Interface with other accelerator subsystems   

In terms of assembly and installation, the magnet block interfaces with other accelerator components can be sorted into the following categories,

(i) Mechanical: attachment points to support stand, fiducial points, attachment and clearance for vacuum chamber.

(ii) Electrical: main coil connections, trim and corrector coil connections, interlock connections.

(iii) Coil cooling water circuit connections.

The mechanical adjustment mechanisms and fiducial points are shown in Fig. 18 ▶. Each magnet block is standing on three support points which are adjustable by ±10 mm in the vertical direction by an M36 thread. The longitudinal and the two sideways adjustment mechanisms are identical in design, consisting of a push block attached on the magnet bottom which is acted on by an M10 adjustment screw from one side and spring washers from the other side, providing ±10 mm adjustment range. The fiducial targets are 1.5" spheres located in conical holes which are machined directly in the yoke top block. They are located directly above the three vertical adjusts, and the two sideways adjusts, above the contact point between the push block and the adjustment screw.

In each magnet block, the vacuum chamber assembly is attached at the beam position monitor house, which is bolted down directly on a precision-machined mating surface in the yoke bottom, with a guiding slot for the sideways position. The vacuum chambers are also vertically supported at brackets mounted on the outer side of the magnet block. All vacuum chamber cooling water leads are routed to the outer side of the magnet block. Fig. 19 ▶ shows the vacuum chamber inside the U1 magnet block, which is designed to provide clearance both for the stored electron beam pipe and the photon beam pipe.

The electrical and water cooling connections are located on the inner side of the magnet blocks, see the example photograph in Fig. 20 ▶. The design is made to allow for easy dis­assembly/reassembly of the magnet block top/bottom halves when installing the vacuum chamber. The coil exit leads are routed through cut-outs on the inner side of the yoke bottom/top blocks, series connected across the midplane^7^, and terminated by flags for cable shoe attachment, located so as to receive power supply cables coming from below. The coil cooling circuits are connected to separate manifolds for the bottom and top halves, which are connected to the main cooling water distribution at one end of each manifold pipe. The different trim windings and corrector coils are wired to one centrally located terminal strip per magnet block half, which are series connected across the midplane by plug-in contacts to allow easy disassembly/reassembly, and connected to power supplies at plug-in contacts on the bottom half. Thermoswitches and limit switches^8^ are also wired to one terminal per block half and connected from top to bottom through a plug-in contact, and connected to the interlock and control systems at d-sub contacts on the magnet block bottom half.

Each magnet element also has its main coils connected to a ‘shunt board’ at the magnet block inner side (see Fig. 20 ▶). The shunt board is a printed circuit board which for each magnet has a set of resistors parallel connected to the magnet main coils. Each resistor is series connected with a plug-in contact so that each resistor circuit is open unless the plug is put in place. By selecting which plugs to put in, a chosen fraction of the current coming from the power supply is shunted through the resistors. This allows for passively tuning the individual magnets to have the same strength within each family.

## 6. Prototype   

A prototype magnet block corresponding to an early version of the M1 magnet block design was manufactured and characterized in 2010/2011 (Johansson *et al.*, 2011 ▶). Apart from measurement results, the prototype also provided practical input to the mechanical design, such as, for example, establishing the tolerance and reference planes scheme for the yoke block drawings (see §4), the guide blocks (see, for example, Fig. 11 ▶), having the quad pole roots machined out of the iron block, coil exit leads geometries, having tolerances on pole shape in assembled state for sextupole and octupole yoke halves instead of on individual parts (see Fig. S2), *etc*.

At the time of writing (spring 2014), the prototype magnet tests are the only investigation that has been performed on cross-talk phenomena in the MAX IV 3 GeV ring magnet blocks. The cases for which Hall probe data were documented^9^ are listed in Table 7 ▶, as max − min of logged field over the applied range of current. The applied current and Hall probe polarities, and in some cases even the Hall probe location, were not documented. So, with the data at hand, in some of these cases it is not possible to distinguish between fringe field overlap and cross-talk through the return yoke.^10^

In Table 7 ▶, several of the field values are stated with a plus/minus range, coming in part from the data being noisy, and also from the response not being equal on powering up and powering down the causing magnet. In such cases better accuracy and more repeats would have been useful to characterize the behaviour.

For the QDend(DIPm) case, a rotating coil measurement and an *Opera3D* simulation were also made, indicating a cross-talk of ∼10 G, 2 T m^−2^ (rotating coil) or 4 G, 3 T m^−2^ (*Opera3D*) in QDend from DIPm at full current (Johansson *et al.*, 2011 ▶), which are of the same order of magnitude as the Table 7 ▶ Hall probe result.

The cross-talk from the dipole into the adjacent magnet elements was considered acceptable since it is a static effect (P. Fernandes Tavares, private communication), but since the data at hand have uncertainties and not all cases are documented, from a magnet design perspective it would be interesting to perform a full characterization of a few actual production series magnet blocks.

## 7. Specification and procurement   

The production of the MAX IV magnet blocks is entirely outsourced to industry, as build-to-print contract where the suppliers are responsible for meeting mechanical tolerances and MAX-lab is responsible for the magnetic field properties. The suppliers also perform all magnetic field measurements.

For each magnet block type, a full set of manufacturing drawings was made by MAX-lab. These, together with the technical specification document (Anderberg *et al.*, 2011 ▶), are the basis for the MAX IV magnet block production. The technical specification gives supporting instructions for production which are not stated on the drawing sheets, such as material and heat treatment specifications, serial number marking, painting, *etc*. It also defines quality assurance, *i.e.* mechanical, electrical, *etc*, and the field measurements. Some of the key specifications are listed below.

**Figure 3:**
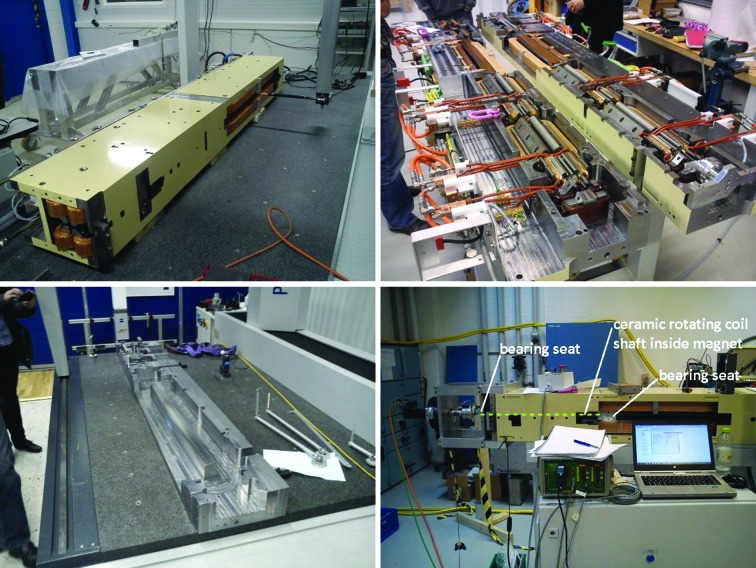
Photographs of MAX IV magnet blocks at different production stages. Top left: U5 magnet undergoing Hall probe measurement. Top right: M1 magnet block top half and bottom half side by side. Bottom left: U4/U5 yoke bottom half undergoing 3D mechanical measurement. Bottom right: M1 magnet undergoing rotating coil measurement.

**Figure 4:**
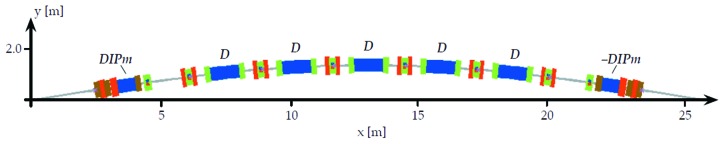
Achromat schematic with placement of dipoles indicated.

**Figure 5:**
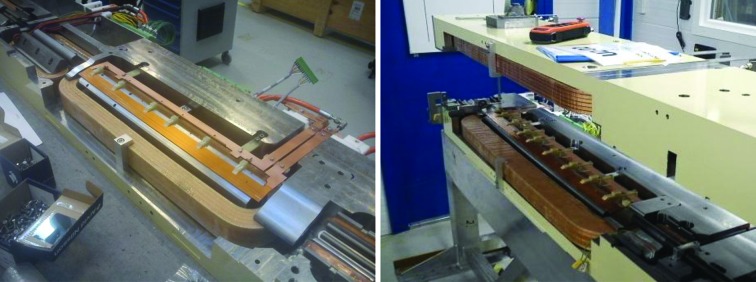
Left: M2 magnet block bottom half being assembled; close up of dipole (DIPm). Note the screw holes for the loose soft end pole part at the far end of the dipole. Right: U2 magnet block with the top half lifted up, exposing the bottom half of the dipole (DIP).

**Figure 6:**
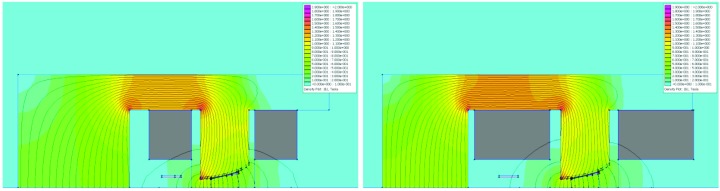
2D simulation results: field distribution in DIP and DIPm magnet cross sections. The colour scale goes from 0 to 2 T. The main coil cross section is marked in grey. The field is highest over the inner leg of the main coil, ∼1.3 T. There is also local saturation of ∼1.8 T at the inner corner of the pole profile. Left: DIPm at NI = 6135 A, with *B* = −0.53334 T, *B*′ = 8.74833 T m^−1^ and *B*′′/2 = 1.41 T m^−2^ [by quadratic fit to simulated *B*_*y*_(*x*) over *x* = ±11 mm]. Right: DIP at NI = 6120 A, with *B* = −0.52831 T and *B*′ = 8.65569 T m^−1^ [by linear fit to simulated *B*_*y*_(*x*) over *x* = ±11 mm].

**Figure 7:**
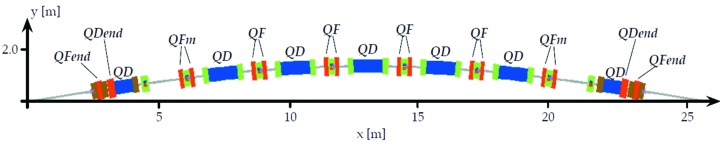
Achromat schematic with placement of quadrupoles indicated. QD refers to the defocusing gradient in DIP and DIPm.

**Figure 8:**
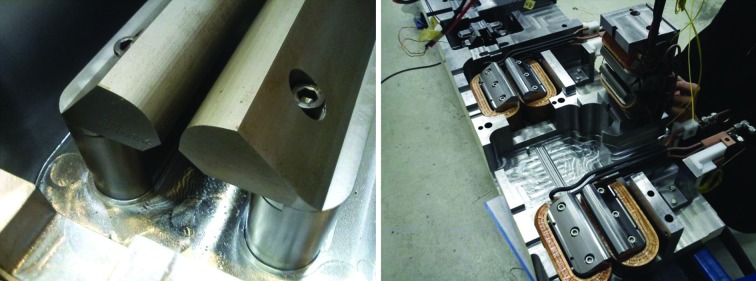
Left: M1 yoke bottom with QDend pole tips mounted on pole roots. Right: U4 magnet block top half being assembled. An SFo sextupole half is about to be installed between the two QF quadrupoles.

**Figure 10:**
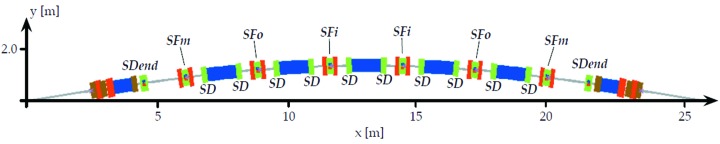
Achromat schematic with placement of sextupoles indicated.

**Figure 11:**
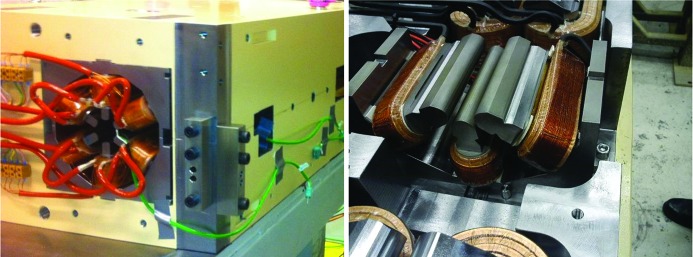
Left: M1 magnet block view from exit side showing SDend sextupole (photograph C. Hansen, Danfysik A/S). Note the guide blocks mounted on the reference planes. Right: SFm sextupole half in U1 magnet block. Note the trim windings on each coil.

(i) ‘All dimensions with tolerances smaller than 0.1 mm must be measured and recorded.’ The tolerances themselves are stated on the drawing sheets using standard geometric dimensioning and tolerancing symbols (see, for example, Figs. S2 and S3). It follows from this specification that every yoke bottom and yoke top block, and every loose yoke piece, must be measured in a three-dimensional CMM.

(ii) ‘Tolerances of ±0.01 and ±0.02 mm are frequently requested on the drawings. These tolerances have been chosen as probably the best practical result attainable. The machining shall aim at reaching these tolerances, but minor and few deviations up to 0.03 mm may be accepted after discussion with MAX-lab.’ See further discussion below.

(iii) ‘For the yoke tops (drawing numbers 3.M1_DMP004, 3.M2_DMP006, 3.U3_DMP004, 3.U4_DMP004 and 3.Ux_DMP010), the fiducial positions must also be measured and recorded. The fiducial positions are defined as the centre of a 38.1 mm (1.5") sphere located in the five Ø30 mm conical holes.’^11^ Together with the yoke top drawings, this instruction defines that the fiducial target locations shall be measured in a three-dimensional CMM, relative to the yoke top reference surfaces.^12^

(iv) Hall probe field measurements, in each dipole: ‘Complete field map: *x* = ±15 mm, with Δ*x* = 1 mm and *s* = 0 to 754.24/1223.78 mm with Δ*s* = 5 mm, giving a total = *ca* 4700/7600 points.’ And in each quadrupole ‘Transverse lines: *x* = ±15 mm, with Δ*x* = 1 mm. At three different longitudinal positions’ through the small inspection ports in the outer yoke which can be seen for example in Fig. 3 ▶.

(v) The specification states that for the Hall probe measurements, the coordinate system shall be aligned relative to the yoke block reference surfaces, but no explicit tolerance is given for the alignment.

(vi) Rotating coil measurement in each quadrupole, sextupole, octupole and corrector, presenting integrated strength of the main term in T, T m^−1^, T m^−2^ or T mm and harmonic content (normal and skew terms) up to *n* = 20, at a reference radius of 10 mm. Measurement coil lengths are specified for the different magnet types.^13^

(vii) Alignment for the rotating coil measurements is specified as that ‘the rotation axis shall be within ±0.1 mm of the nominal trajectory, defined relative to the reference surfaces’.

The field measurements section of the specification document gives some further supporting instructions such as set cycle, current levels, *etc.*, and also defines a series of repeatability checks to be performed at the start of the productions series, including disassembly and reassembly of magnet blocks. But, essentially, the specification does not give a step-by-step recipe for how to perform the field measurements; rather it lists the measurement data that shall be presented to MAX-lab for each magnet block, leaving it up to the supplier to figure out how to perform the field measurements. This included solving how to access the different magnet elements inside the magnet blocks for rotating coil measurements.

It follows from the above-listed specifications that the fiducialization concept for the MAX IV magnet blocks is to align the magnets relative to the mechanical reference surfaces on the yoke blocks, with the alignment of individual magnet elements within the magnet blocks given by the mechanical tolerances, which are related to the same reference surfaces, *i.e.* field measurement of the magnetic centre locations relative to the fiducial target locations was not specified to be performed with high accuracy.

The sourcing of the magnet block production was a standard public procurement procedure, with the call for tender being for fully assembled and tested magnet blocks, according to the assembly drawings listed in Table 5 ▶ and the technical specification, for a fixed price and a fixed delivery time. The full set of manufacturing drawings was sent out with the call for tender. Separate quotes were requested for three different ‘packages’ of magnet blocks:

(*a*) 20 pieces (pcs) M1 magnet blocks and 20 pcs M2 magnet blocks;

(*b*) 40 pcs U1/U2 magnet blocks and 40 pcs U4/U5 magnet blocks;

(*c*) 20 pcs U3 magnet blocks;

(*Cf.* description of magnet block types in §4.^14^)

In the yoke bottom and yoke top block drawing versions sent out with the call for tender, the critical tolerances listed in §4 above were twice as hard, ±0.01 mm for guiding surfaces, 0.02 mm for dipole profile and mid-plane flatness, and 0.01 mm for reference surfaces. These tolerances were relaxed to present values during contract negotiations. The other major changes that occurred during the procurement phase was that the ferrite version of the correctors was replaced (see §3.5) and that the mechanical tolerance concept for sextupoles and octupoles was changed from being defined on the individual parts to being defined per yoke half assembly, meaning a harder requirement for the suppliers (see §6).

Contracts for the three packages listed above were awarded to two separate suppliers:

(i) 60 pcs M1, M2 and U3: Danfysik A/S (Danfysik A/S, Taastrup, Denmark);

(ii) 80 pcs U1, U2, U4 and U5: Scanditronix Magnet AB (Scanditronix Magnet AB, Vislanda, Sweden).

At the time of writing (spring 2014), the production series of parts, such as yoke blocks, coils, *etc.*, is near completion, and assembly/testing/field measurement of complete magnet blocks is roughly halfway through, with delivery to MAX-lab ongoing. Some results from the production series are presented in §8 and §9 below.

### 7.1. Iron blocks   

Before the complete magnet blocks were procured, a separate procurement was made for iron blocks to be used for the yoke bottom and yoke top blocks. The call for tender and subsequent contracts for the magnet blocks were then written such that the raw material for the yoke bottom and top machining was free-issued by MAX-lab (Table 8 ▶).

The specification given for the iron blocks was chemical-composition maximum content stated as C 0.010%, Mn 0.060%, P 0.005%, S 0.003%, N 0.005%, Cu 0.030%, Co 0.005%, Sn 0.005%. Furthermore, a heat treatment was performed on all iron blocks, specified as heating to 1003 K and hold for 10 h, then cooling at a controlled rate of 10 K h^−1^ down to 573 K, followed by free cooling. The purpose of the heat treatment is to improve the saturation magnetization through promoting grain growth.^15^

For the other loose yoke pieces (quad pole tips *etc*), the magnet suppliers provided the material themselves, with the same chemical composition and heat treatment stated in the magnet block technical specification.

## 8. Mechanical measurement results from the production series   

As described in the previous sections, with the MAX IV magnet block concept, field quality and alignment of magnet elements within each block depend on the machining accuracy of the yoke bottom and yoke top blocks, and the loose yoke parts that are mounted in the blocks. Presented in this section are mechanical measurement results for the large yoke blocks.

As described in §7 above, the specification implies verification by 3D coordinate measurement machine for all yoke parts. Typically, 3D CMM data are presented as a measurement report containing a calculated value for each dimension, difference between calculated value and nominal value, and an indication of whether or not the difference is within tolerance. For the yoke bottom and yoke top block measurements, we have also had the suppliers provide us with the underlying point data. As mentioned in §4 above, the yoke block critical dimensions can be sorted into vertical guiding, sideways guiding, dipole profile and reference surfaces. Point data for an example yoke block from the middle of the production series sorted in these categories is shown in Figs. 22 to 26, with the 3D CAD model of the yoke block shown in Fig. 21 ▶ for comparison.

Some comments on the Figs. 22–26 3D measurement data are listed below,

(i) The data points on the mid-plane (Fig. 22 ▶) have average *y*(*z*) = 0 and slope *y*(*z*) = 0, indicating that the measurement evaluation coordinate system is correctly aligned.^16^ The alignment of the *x*,*z*-plane is made as best fit to all the data points on the mid-plane.

(ii) The data points on the sideways reference planes (Fig. 23 ▶) both have *x*(*y*) = 0 at *y* = 0, indicating correct alignment. The *z*-axis is aligned by creating reference A and reference B planes as best fit to the data points on these surfaces and setting *x* = 0 at the intersection between these planes and the *x*,*z*-plane.

(iii) The data points on the longitudinal reference plane (Fig. 23 ▶) have *z*(*y*) = 0 at *y* = 0, indicating correct alignment. The origin is located by creating a reference C plane as best fit to the data points taken on this surface and setting *z* = 0 at the intersection between this plane and the *x*,*z*-plane.^17^

(iv) The Figs. 22 ▶ ▶ ▶ ▶–26 ▶ example data are representative of the accuracy that has been achieved for the MAX IV yoke blocks in that there are always at least a few of the critical surfaces which are close to, or above, the ±0.02 mm tolerance level. For example, in Fig. 23 ▶ the reference B surface is clearly above its 0.02 mm perpendicularity tolerance. However, since the surface is tilting outwards, the contact point between the bottom/top yoke blocks and the guide block is not affected, so this deviation has no impact on performance. This exemplifies how we have applied the ‘minor and few deviations up to 0.03 mm may be accepted after discussion with MAX-lab’-specification, by a consequence analysis to determine whether or not corrective action is needed.

(v) The sideways and vertical point data in the Figs. 24 ▶–25 ▶ example are typical in that the deviations tend to correlate between consecutive guiding surfaces, and between left and right poles belonging to the same magnet, *i.e.* there are typically not deviations of opposite sign over the full tolerance span between consecutive elements, but rather there is tilt, or offset, or curvature over the whole length of the yoke block.

At the time of writing (spring 2014) the yoke bottom and yoke top block production series is close to completion, with achieved accuracy generally within the ±0.02 mm tolerance.

## 9. Field measurement results from the production series   

At the time of writing (spring 2014) we are roughly halfway through the magnet production series, meaning that a large amount of Hall probe and rotating coil magnetic field measurements have been performed, and several aspects of magnet performance have been analyzed. In this section we will focus on one aspect that is of particular conceptual importance, namely alignment of magnet centres within magnet blocks.

As described in §4 and §7, the MAX IV magnet block design assumes that the magnetic centre location for each magnet element is given by the mechanical locations of the pole surfaces, *i.e* that the alignment accuracy within each block is given by the mechanical tolerances of the yoke bottom/yoke top blocks, the loose quad pole tips and the sextupole/octupole yoke halves. And as described in §7, the technical specification made no attempt to have the magnetic centre locations relative to the mechanical reference surfaces measured with any high accuracy. However, with the field measurement data that have been obtained, we have tried to draw some conclusions, as described below.

For the issue of how to access the magnet elements inside the magnet blocks for rotating coil measurement, both magnet suppliers chose solutions with a long rotating shaft containing several measurement coils, located at each magnet element. Example photographs are shown in Figs. 3 ▶ and 27 ▶. The Danfysik solution was a ceramic shaft with tangential measurement coils, held by bearings at each end. The bearing locations are given by a fixture resting on the dipole profile at the inner end and a frame mounted on magnet block external surfaces at the outer end. Danfysik built three different rotating coil assemblies, with different measurement coils for measuring the following magnet elements:

(i) ‘M1/M2 long’: corr *x*/*y*–OXX–QFend–OXY–QDend.

(ii) ‘M1/M2 short’: OYY–corr *y*/*x*–SDend.

(iii) ‘U3’: QF–SFi–QF–corr *y*/*x*–SD.

Compare with Table 10 showing the magnet element order within the magnet blocks. The Scanditronix Magnet solution was a carbon fibre shaft with radial measurement coils. Two different rotating coil assemblies were built for the U1, U2, U4 and U5 magnet blocks, containing different measurement coils for the following magnet elements:

(i) ‘long’: QFm–SFm–QFm/QF–SFo–QF.

(ii) ‘short’: SD.

*Cf.* Table 10. The ‘short’ rotating coil assembly is used for measuring the single SD sextupole at the end of the U1/2/4/5 magnet block. Both rotating coil assemblies have bearings at each end, but the ‘long’ rotating coil also has a bearing at the centre to counteract sag. The bearing locations are given by milled-out slots in the yoke bottom block that were added by Scanditronix Magnet.

Both for Danfysik and Scanditronix Magnet, the rotating axis positioning accuracy relative to the mechanical reference surfaces is given by a tolerance chain consisting of mounting surfaces/slots on the yoke blocks, and bearing seat parts, which is within the specified ±0.1 mm, but not good enough to verify the supposed alignment accuracy of 25 µm r.m.s. relative to the reference surfaces. Note, however, that, by having several measurement coils on the same rotating shaft, the magnet elements that are measured with the same rotating coil assembly in the same magnet block are measured in a common local coordinate system. So although the absolute location relative to the reference surfaces is only known to within ±0.1 mm, the relative alignment of the consecutive magnets can be seen with high accuracy.

From the rotating coil measured harmonic content, the horizontal and vertical offset, d*x* and d*y*, between the rotating axis and the magnetic centre is calculated as d*x* ≃ (*r*_0_/*n*)*B*_*n*–1_/*B*_*n*_, d*y* ≃ (*r*_0_/*n*)*A*_*n*–1_/*B*_*n*_ (Russenschuck, 2010 ▶), where we have let *B*_*n*_ denote the main harmonic term, all of 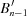 and 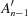 is assumed to be feed-down, and *r*_0_ is the radius at which the harmonic content is presented. Example calculated offsets for a few measurements performed with the Danfysik ‘M1/M2 long’ rotating coil assembly are shown in Fig. 28 ▶, plotted as a function of longitudinal position of the measured magnet, where *z* = 0 is the location of the longitudinal reference surface of the M1 yoke blocks.

As seen in Fig. 28 ▶, there are offsets of up to 0.1 mm between the rotating coil axis and the magnets, but within each magnet block the relative alignment of these four consecutive magnet elements appears to be much better, especially in the horizontal direction. The relative alignment is obtained by subtracting linear fits from d*x*(*z*) and d*y*(*z*). Calculated relative alignment for the Fig. 28 ▶ data is shown in Fig. 29 ▶.

As seen in Fig. 29 ▶, for these six example M1 magnet blocks, the relative alignment of magnet elements OXX, QFend, OXY and QDend is within +5–6 µm in the horizontal direction, which is well within the assumed alignment accuracy, and within +17–24 µm in the vertical direction, which is worse, but still within the assumed accuracy. For the vertical offsets it is possible that we are seeing the combined effect of individual magnetic centre deviations and rotating shaft sag.

The same analysis has been performed for all obtained ‘M1/M2 long’ rotating coil measurements, which at the time of writing is 38 magnet blocks, almost the full production series of these types. Results are shown in Fig. 30 ▶ and Table 9 ▶.

The same analysis is not possible for the magnet elements on the other side of the M1/M2 magnet blocks, since there are only two magnets for which the magnetic centre can be defined, namely OYY and SDend. For the unit-cell magnet blocks, there are four consecutive magnet elements which are measured with the same rotating coil assembly. However, for the U1, U2, U4 and U5 magnet blocks, only three of these magnets can be considered to have been measured in the same local coordinate system, since the Scanditronix Magnet ‘long’ rotating coil assembly has an intermediate bearing between the inner quadrupole and the corrector pair (see Fig. 27 ▶), and the rotation axis can only be considered to be straight between two bearings. So, for the U1, U2, U4 and U5 magnet blocks, relative alignment has been calculated for three consecutive magnets, and for the U3 magnet blocks relative alignment has been calculated for four consecutive magnet elements. At the time of writing, this analysis has been performed for 34 U1, U2, U4 and U5 magnet blocks. Results are listed in Table 9 ▶. For U3 magnet blocks we do not have enough measurements yet to present any meaningful statistics.

**Figure 12:**
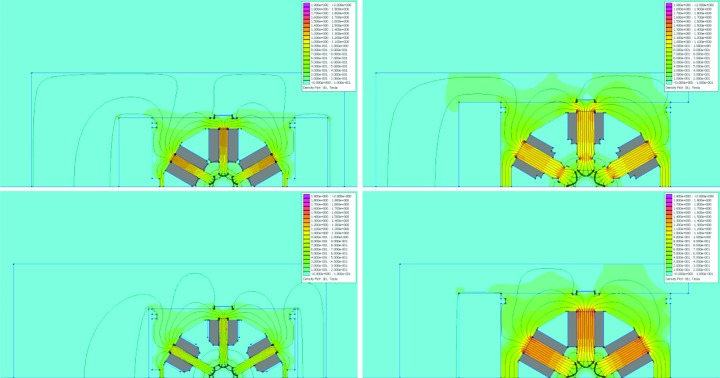
2D simulation results: field distribution in sextupole magnet cross sections. The colour scale goes from 0 to 2 T. The main coil cross section is marked in grey. The field is highest in the bottom of the pole root, between 0.9 and 1.4 T for the different variants. Top left: SDend at NI = 969.8 A, with *B*′′/2 = 1769.59 T m^−2^. Top right: SFm at NI = 969.8 A, with *B*′′/2 = −1710.67 T m^−2^. The pole side has a cut-out to provide clearance for the vacuum chamber. Bottom left: SD at NI = 664.4 A, with *B*′′/2 = 1223.45 T m^−2^. Bottom right: SFi at NI = 1179 A, with *B*′′/2 = −2094.90 T m^−2^.

**Figure 13:**
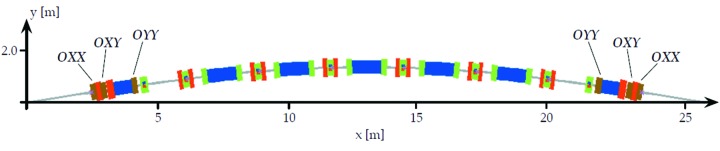
Achromat schematic with placement of octupoles indicated.

**Figure 14:**
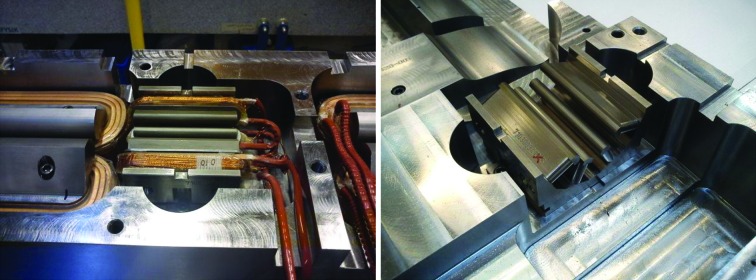
Left: OXY octupole half in M1 magnet block between QFend and QDend quadrupoles. Note the trim windings on each coil. Right: OYY yoke half mounted in M1 yoke bottom.

**Figure 16:**
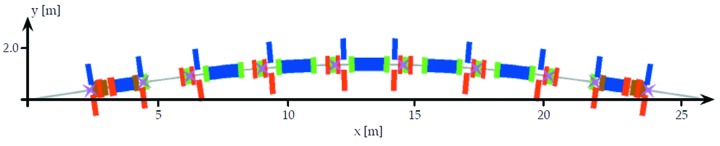
Achromat schematic with placement of beam position monitors indicated as magenta crosses, and horizontal/vertical correctors indicated by blue and red strips. Note that this schematic has two vertical correctors in matching cell 1, but one of these (between OYY and SDend) was removed in order to allow passage of the photon beam pipe.

**Figure 17:**
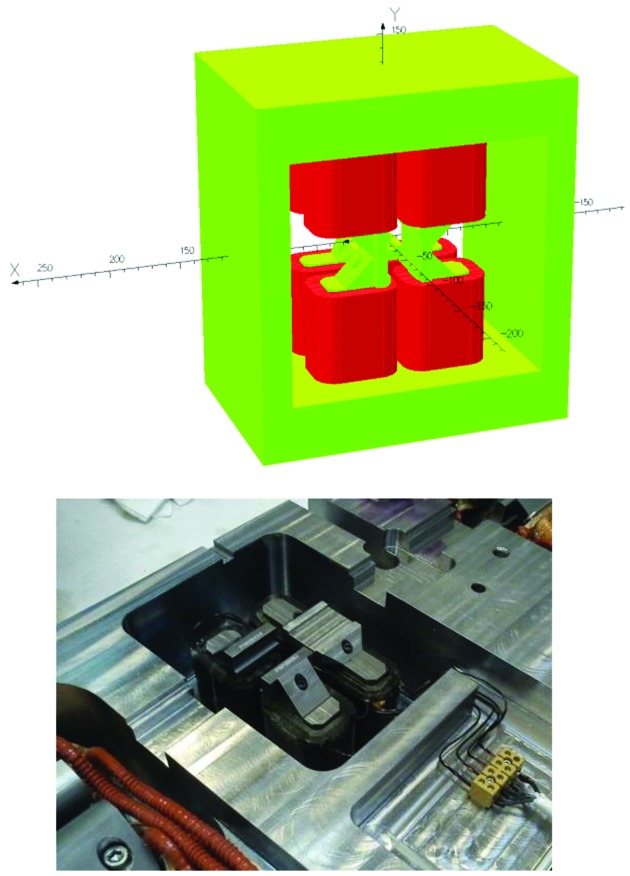
Top: *Opera3d* model of corrector pair, view from vertical corrector side [no 2D simulation was made since the correctors are short and located close together; the corrector pair was designed using *Opera3d* (http://operafea.com/)]. The coil current directions are set so as to generate a horizontally directed field between the left and right poles. The simulated integrated strength is ∼3.8 T mm (0.38 mrad) at NI = 1280 A both for horizontal and vertical. Bottom: assembled U3 magnet block bottom half showing one corrector pair.

**Figure 18:**
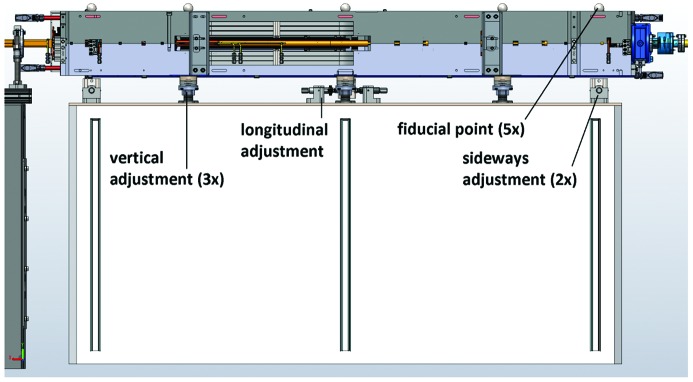
Achromat 3D CAD assembly: M1 magnet block on support stand, view from outer side.

**Figure 19:**
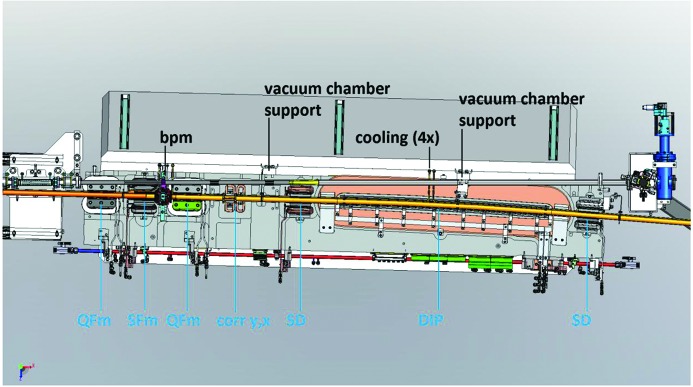
Achromat 3D CAD assembly; U1 magnet block view from above with magnet block top half hidden. Note that the sextupole magnets are designed such that the photon beam pipe passes on the inside of the SFm return yoke and on the outside of the SD return yoke.

**Figure 20:**
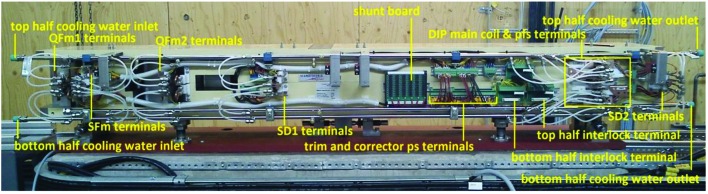
U1 magnet block with plastic cover removed; view from inner side.

**Figure 21:**
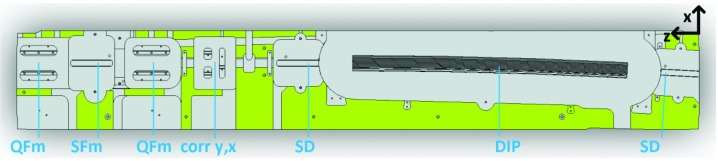
Uc1–2 yoke bottom 3D CAD model: view from above with Fig. 22 ▶ to Fig. 26 ▶ 3D CMM data origin and axis directions indicated (+*y* is toward the viewer). The mid-plane (reference D) is marked green.

**Figure 22:**
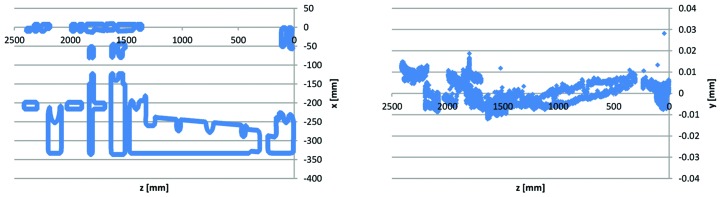
3D CMM point data for Uc1–2 yoke bottom #13622-53: mid-plane (reference D) points *x*(*z*) and and *y*(*z*). *Cf.* mid-plane geometry in Fig. 21 ▶.

**Figure 23:**
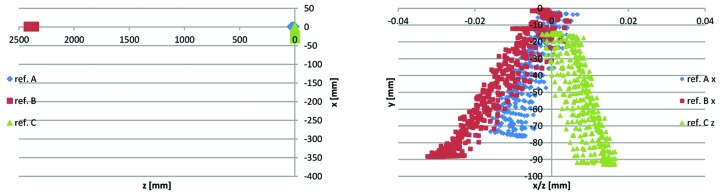
3D CMM point data for Uc1–2 yoke bottom #13622-53: reference planes A, B and C points. Left: *x*(*z*). Right: planes A and B *x*(*y*) and plane C *z*(*y*).

As seen in Table 9 ▶, the measured relative alignment of three or four consecutive magnet elements is well within the assumed alignment accuracy. However, since the absolute location of the rotating coil is not known with high accuracy, the measurement does not indicate directly if these three or four consecutive magnet elements are well aligned relative to the other elements within the same magnet block. Looking at the example rotating coil measurement results in Fig. 28 ▶, with offsets of up to 0.1 mm between the rotation axis and the magnet centres, the situation could be either that the magnets are well aligned and what we are seeing are positioning errors of the rotating coil, or that the rotating coil axis is well aligned and that the magnets, although well aligned relative to each other locally, are misaligned by up to 0.1 mm relative to the magnet block reference surfaces. Assuming that the magnetic centres agree with the mechanical centres, the mechanical measurement data would rule out the latter case, with yoke bottom and yoke top blocks and loose yoke parts having been measured to be within their mechanical tolerances. On the other hand, the case could be that magnetic centres are not agreeing with mechanical centres. Such a mismatch would have to be caused by differences or anisotropy of the magnetization properties between different yoke parts or different regions of the yoke. Considering that consecutive magnet elements have been measured to be locally aligned to within the level shown in Table 9 ▶, the anisotropy would have to be in the yoke parts that are common to all magnet elements, which are the large yoke bottom and yoke top blocks. But considering also that the sextupoles and octupoles have almost all of their return flux going through their own return yoke (see Figs. 12 ▶ and 15 ▶), such differences in magnetization properties would have to exist also in these yoke parts, with direction and magnitude equal to any in the large yoke blocks. Such a coincidence we consider to be unlikely beyond reasonable doubt.

**Figure 24:**
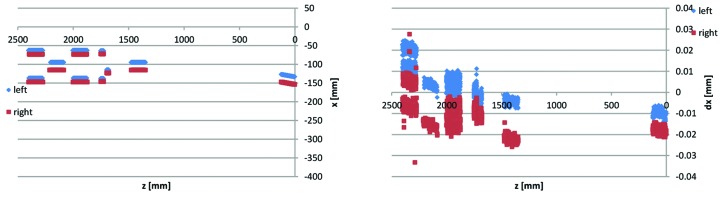
3D CMM point data for Uc1–2 yoke bottom #13622-53: sideways guiding slot points *x*(*z*) and d*x*(*z*).

**Figure 25:**
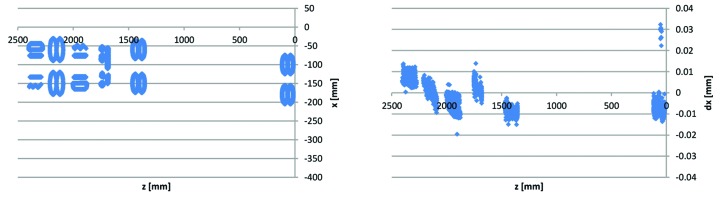
3D CMM point data for Uc1–2 yoke bottom #13622-53: vertical guiding surface points *x*(*z*) and d*x*(*z*).

**Figure 26:**
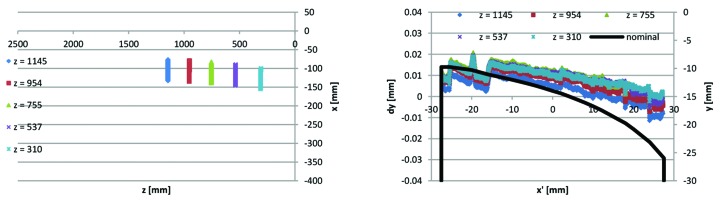
3D CMM point data for Uc1–2 yoke bottom #13622-53: dipole profile points measured at five longitudinal locations. Left: *x*(*z*); right: measured d*y*(*x*) and nominal *y*(*x*) (*cf*. Fig. 6 ▶).

**Figure 27:**
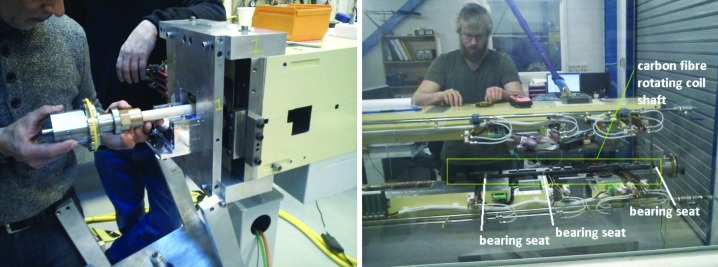
Left: Danfysik ‘M1/M2 short’ rotating coil assembly being inserted into an M1 magnet block. The frame with the outer bearing seat is mounted on the magnet. See also Fig. 3 ▶. Right: Scanditronix magnet ‘long’ rotating coil assembly being installed in a U4 magnet block. The top half is lifted up to allow access to the bearing seats. (See also Fig. 5 ▶, where part of the rotating coil shaft and one bearing holder can be seen.)

**Figure 28:**
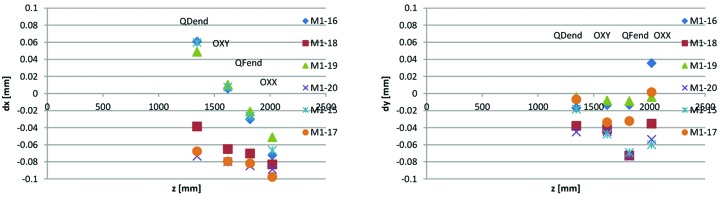
Rotating coil measurement results: offset d*x* and d*y* between rotating axis and magnetic centre calculated from harmonic content feed down, as a function of magnet element longitudinal position *z* along magnet block. For magnet element OXX, QFend, OXY and QDend in M1 magnet blocks serial number 15–20.

**Figure 29:**
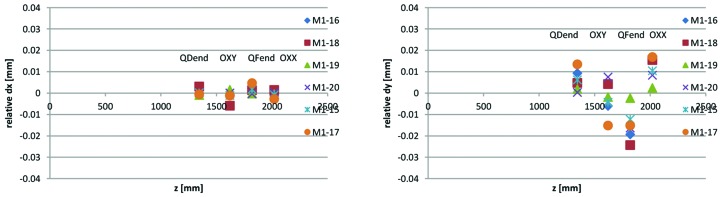
Rotating coil measurement results: relative magnetic centre locations d*x* and d*y* between consecutive magnet elements calculated from harmonic content feed down, as a function of longitudinal position *z* along magnet block. For magnet element OXX, QFend, OXY and QDend in M1 magnet blocks serial number 15–20.

**Figure 30:**
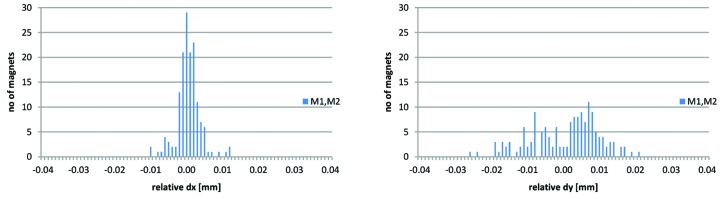
Rotating coil measurement results: histograms of relative alignment d*x* and d*y* between consecutive magnet elements OXX, QFend, OXY and QDend for 38 measured M1 and M2 magnet blocks.

Thus we conclude that the Table 9 ▶ results can be taken as an upper bound on the combined effects of tolerance stack-up of different yoke parts assembled together, and any differences between mechanical and magnet centres. Noting that the Table 9 ▶ results are for rotating coil measurements over a limited part of the total magnet blocks lengths, a conservative estimate would be to assume that over the whole magnet block length the relative alignment between all magnet elements is equal to the Table 9 ▶ results plus ±0.02 mm, the tolerance level applying to the full length of the yoke blocks. This would be roughly equal to the MAX IV DDR requirement of 25 µm r.m.s. with a 2σ cut-off (Leemann, 2010*a* ▶), indicating that from the perspective of magnet-to-magnet alignment we are on track for achieving the design performance for the MAX IV 3 GeV storage ring. Potentially, since mechanical deviations within the yoke blocks ±0.02 mm tolerance level are typically correlated among consecutive elements (as exemplified by the Figs. 24 ▶–25 ▶ data), a detailed statistical analysis of the obtained mechanical measurement data could reduce the estimated magnet-to-magnet alignment further.

## Supplementary Material

MAX IV drawings Figs. S1, S2 and S3: 3.U3_DMA002 `Uc3, assy' sheet 1/6; 3.Ux_DMP009 `Yoke bottom, Uc1-2' sheet 1/3; 3.Mx_DMP002 `Q-pole, Mc', the pole tip drawing for QFend and QDend.. DOI: 10.1107/S160057751401666X/xe5010sup1.pdf

## Figures and Tables

**Figure 9:**
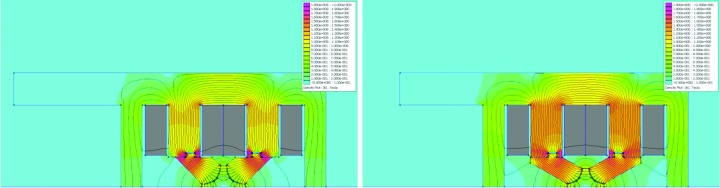
2D simulation results: field distribution in QFend and QF magnet cross sections. The colour scale goes from 0 to 2 T. The coil cross section is marked grey. Left: QFend at NI = 2273.7 A, with *B*′ = −33.7908 T m^−1^. The field is highest around the pole tip/pole root mating surface, ∼1.5 T. There is also local saturation of ∼1.8 T at the guiding slot. Right: QF at NI = 3010.8 A, with *B*′ = −41.9869 T m^−1^. The field is ∼1.3–1.5 T throughout the pole tip and pole root, with local saturation of ∼1.8 T at the guiding slot.

**Figure 15:**
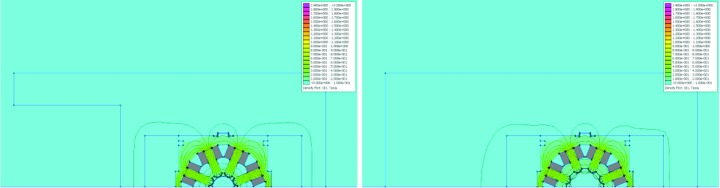
2D simulation results: field distribution in octupole magnet cross sections. The colour scale goes from 0 to 2 T. The main coil cross section is marked in grey. Left: OXY at NI = 315.9 A, with *B*′′′/6 = −63285.1 T m^−3^. The field is highest in the bottom of the pole roots, ∼0.6 T. Right: OYY at NI = 592.2 A, with *B*′′′/6 = 26937.2 T m^−3^.

**Table 1:** Dipole lattice elements with corresponding field strengths at 3 GeV (from Appendix *A* [App appa], Table 10 ▶) Both DIPm and DIP consist of 12 slices. Length and bend angle stated in this table are sum of slices; *k*, *B* and *B*′ are for the central slice.

Element	Segment	Total no. (pcs)	*l* (m)	*t* (°)	*k* (m^−2^)	*B* (T)	*B*′ (T m^−1^)	Comment
DIPm	MC1,2	40	0.75424	1.5	−0.86146	−0.52405	8.62207	12 slices
DIP	UC1–5	100	1.22378	3	−0.85998	−0.52405	8.60722	12 slices

**Table 2:** Quadrupole lattice elements with corresponding field strengths at 3 GeV (from Appendix *A* [App appa], Table 10 ▶)

Element	Segment	Total no. (pcs)	*l* (m)	*k* (m^−2^)	*B*′ (T m^−1^)
QFend	MC1,2	40	0.25	3.656234	−36.5939
QDend	MC1,2	40	0.25	−2.507705	25.0987
QFm	UC1–5	80	0.15	3.776156	−37.7941
QF	UC1–5	160	0.15	4.030907	−40.3438

**Table 3:** Sextupole lattice elements with corresponding field strengths at 3 GeV (from Appendix *A* [App appa], Table 10 ▶)

Element	Segment	Total no. (pcs)	*l* (m)	*k* (m^−3^)	*B*′′/2 (T m^−2^)
SDend	MC1,2	40	0.1	−170	1701.47
SFm	UC1,5	40	0.1	170	−1701.47
SD	UC1–5	200	0.1	−116.4139	1165.14
SFo	UC2,4	40	0.1	174	−1741.50
SFi	UC3	40	0.1	206.7070	−2068.85

**Table 4:** Octupole lattice elements with corresponding field strengths at 3 GeV (from Appendix *A* [App appa], Table 10 ▶)

Element	Segment	Total no. (pcs)	*l* (m)	*k* (m^−4^)	*B*′′′/6 (T m^−3^)
OXX	MC1,2	40	0.1	−1618.313	16197.1
OXY	MC1,2	40	0.1	3250.189	−32529.9
OYY	MC1,2	40	0.1	−1416.59	14178.1

**Table 5:** MAX IV 3 GeV storage-ring magnet block types and corresponding lattice segments (*cf*. Figs. 1 ▶, 2 ▶ and Appendix *A*, Table 10 ▶) The special versions listed are identical in terms of lattice, but have mechanical differences to fit special vacuum chambers in those magnet blocks.

Type	Drawing number	Lattice segment	Total no. (pcs)	*w* × *h* × *l* (mm)	Weight (kg)	Comment
M1	3.M1_DMA002	MC1	19	350 × 256 × 2298.7	1180	
U1	3.U1_DMA002	UC1	19	350 × 256 × 2417.5	1300	
U2	3.U2_DMA002	UC2	20	350 × 256 × 2417.5	1300	
U3	3.U3_DMA002	UC3	20	349.7 × 256 × 3356	1580	
U4	3.U4_DMA002	UC4	20	350 × 256 × 2417.5	1300	
U5	3.U5_DMA002	UC5	19	350 × 256 × 2417.5	1300	
M2	3.M1_DMA002	MC1	19	350 × 256 × 2298.7	1180	
3.01.M1	3.01.M1_DMA002	achr1 MC1	1	350 × 256 × 2298.7	1180	OXX, OXY†, cut-out in yoke blocks
3.01.M2	3.01.M2_DMA002	achr1 MC2	1	350 × 256 × 2298.7	1180	
3.02.U1	3.02.U1_DMA002	achr2 UC1	1	350 × 256 × 2417.5	1300	SFm coils
3.02.U5	3.02.U5_DMA002	achr2 UC5	1	350 × 256 × 2417.5	1300	SFm coils, cut-out in yoke blocks

† 3.01.M1 and 3.01.M2 both have the Ø36 mm aperture OYY type yoke at the OXX and OXY positions to accommodate the wider vacuum chamber after injection, meaning that these OXX and OXY are operated at higher current.

**Table 6:** Yoke block types in the 3 GeV ring, including all special versions

Type	Drawing number	In magnet block	Total no. (pcs)	*w* × *h* × *l* (mm)	Weight (kg)	Comment
Yoke bottom, Mc1	3.M1_DMP003	M1	19	350 × 128 × 2298.7	448	
Yoke top, Mc1	3.M1_DMP004	M1	19	350 × 128 × 2298.7	450	
Yoke bottom, Uc1–2	3.Ux_DMP009	U1, U2	40	350 × 128 × 2417.5	493	
Yoke top, Uc1–2	3.Ux_DMP010	U1, U2	40	350 × 128 × 2417.5	494	
Yoke bottom, Uc3	3.U3_DMP003	U3	20	349.7 × 128 × 3356	618	
Yoke top, Uc3	3.U3_DMP004	U3	20	349.7 × 128 × 3356	617	
Yoke bottom, Uc4–5	3.U4_DMP002	U4, U5	19	350 × 128 × 2417.5	493	
Yoke top, Uc4–5	3.U4_DMP004	U4, U5	19	350 × 128 × 2417.5	494	
Yoke bottom, Mc2	3.M2_DMP001	M2	19	350 × 128 × 2298.7	448	
Yoke top, Mc2	3.M2_DMP006	M2	19	350 × 128 × 2298.7	450	
Yoke bottom, Mc1	3.01.M1_DMP003	3.01.M1	1	350 × 128 × 2298.7	449	Cut-out†
Yoke top, Mc1	3.01.M1_DMP004	3.01.M1	1	350 × 128 × 2298.7	450	Cut-out†
Yoke bottom, Uc4–5	3.02.U5_DMP002	3.02.U5	1	350 × 128 × 2417.5	492	Cut-out‡
Yoke top, Uc4–5	3.02.U5_DMP004	3.02.U5	1	350 × 128 × 2417.5	493	Cut-out‡
Yoke bottom, Mc2	3.01.M2_DMP001	3.01.M2	1	350 × 128 × 2298.7	448	Cut-out†
Yoke top, Mc2	3.01.M2_DMP006	3.01.M2	1	350 × 128 × 2298.7	449	Cut-out†

† Wider cut-out between OXY and QDend for wider vacuum chamber after injection.

‡ Cut-outs after the dipole for diagnostic photon beam pipe.

**Table 7:** Hall probe measurement results: field measured at a fixed position in one magnet element as a function of excitation current in another magnet element, from data files listed by Johansson (2011 ▶) Hall probe position indicated as *s*, *x* (mm), where *s* = 0 is the longitudinal mid-point of the magnet element in question. [This table only contains magnet elements OXX to OYY since the matching cell prototype did not contain the correctors and SDend, *cf.* Appendix *A*, Table 10 ▶. No measurement was documented with the Hall probe in OXY, or with OYY powered.]

		*B* † (G) seen by					
Caused by	NI† (A)	OXX	QFend	OXY	QDend	DIPm	OYY
OXX	219.9		0.2 ± 0.2, at *s*, *x* ≃ −115, ?				
QFend	2187.9	−3 ± 1, at *s*, *x* ≃ 0, 16					
OXY	219.9		0.1 ± 0.1, at *s*, *x* ≃ 115, ?		<0.1 at *s*, *x* ≃ −115, ?		
QDend	1353.3					±0.2 at *s*, *x* ≃ 0, 0	
						±0.2 at *s*, *x* ≃ −250, 0	
						−0.5 at *s*, *x* ≃ −380, 0	
DIPm	11700				−6.8 ± 0.1, at *s*, *x* ≃ 115, 0		−7.3 ± 1, at *s*, *x* ≃ 0, 0
					−9.9 at *s*, *x* ≃ 115, 24		
OYY							

† Hall probe and current polarities not documented.

**Table 8:** Iron blocks purchased by MAX-lab for yoke block machining

Total no. (pcs)	*w* × *h* × *l* (mm)	For yoke block
84	369 × 141 × 2315	M1, M2 yoke bottom and yoke top
168	369 × 141 × 2433	U1, U2, U4, U5 yoke bottom and yoke top
42	369 × 141 × 3370	U3 yoke bottom and yoke top

**Table 9:** Rotating coil measurement results: relative alignment between consecutive magnet elements within the same magnet block

Magnet elements	In magnet block	Evaluated (pcs)	Relative alignment	Minimum (µm)	Maximum (µm)	r.m.s. (µm)	Comment
OXX–QFend–OXY–QDend	M1, M2	38/40	d*x*	−10	12	3	
			d*y*	−27	20	9	Includes rotating coil sag
QFm–SFm–QFm, QF–SFo–QF	U1, U2, U4, U5	34/80	d*x*	−11	12	4	
			d*y*	−18	9	6	Includes rotating coil sag
QF–SFi–QF–SD	U3	–/20	d*x*	–	–	–	
			d*y*	–	–	–	

**Table 10:** MAX IV 3 GeV storage ring lattice elements listed in consecutive order, starting at the centre of a long straight, with corresponding magnetic field strengths at 3 GeV [from Tracy-3 lattice file ‘m4-20111213-420-bare.lat’ (Leemann, 2011*b* ▶)]

Segment	Element	*l* (m)	*t* (°)	*k* (m^−2^)	*k* (m^−3^)	*k* (m^−4^)	*B* (T)	*B*′ (T m^−1^)	*B*′′/2 (T m^−2^)	*B*′′′/6 (T m^−3^)	Comment
LS	…	2.321									
GS											Girder start
MC1	BPM	0.05									
	STR0058	0.058									
	CORR_D	0.025									
	CORR_H	0									Horizontal correction
	2*CORR_D	0.05									
	CORR_V	0									Vertical correction
	CORR_D	0.025									
	STR0021	0.021									
	OXX	0.1				−1618.313				16197.1	†
	STR0025	0.025									
	QFend	0.25		3.656234				−36.5939			
	STR0025	0.025									
	OXY	0.1				3250.189				−32529.9	†
	STR0100	0.1									
	QDend	0.25		−2.507705				25.0987			
	STRx006	0.00608									
	DIPm‡	0.75424	1.5	−0.85998			−0.52405	8.60722			
	OYY	0.1				−1416.59				14178.1	†
	STRx093	0.09268									
	CORR_D	0.025									
	CORR_H	0									Horizontal correction
	3*CORR_D	0.075									
	BPM	0.05									
	STR0020	0.02									
	SDend	0.1			−170				1701.47		
GE											Girder end
S	…	1.302									
UC1	GS										Girder start
	QFm	0.15		3.776156				−37.7941			
	STR0075	0.075									
	SFm	0.1			170				−1701.47		
	STRx013	0.0125									
	BPM	0.05									
	STRx013	0.0125									
	QFm	0.15		3.776156				−37.7941			
	STR0100	0.1									
	CORR_D	0.025									
	CORR_V	0									Vertical correction
	2*CORR_D	0.05									
	CORR_H	0									Horizontal correction
	CORR_D	0.025									
	STRx203	0.20311									
	SD	0.1			−116.4139				1165.14		
	STR0010	0.01									
	DIP‡	1.22378	3	−0.861464			−0.52405	8.62207			
	STR0010	0.01									
	SD	0.1			−116.4139				1165.14		
	GE										Girder end
	STRx403	0.40311									
UC2	GS										
	QF	0.15		4.030907				−40.3438			
	STR0075	0.075									
	SFo	0.1			174				−1741.50		
	STRx013	0.0125									
	BPM	0.05									
	STRx013	0.0125									
	QF	0.15		4.030907				−40.3438			
	STR0100	0.1									
				… identical to UC1							
											
UC3	GS										
	QF	0.15		4.030907				−40.3438			
	STR0075	0.075									
	SFi	0.1			206.7070				−2068.85		
	STRx013	0.0125									
	BPM	0.05									
	STRx013	0.0125									
	QF	0.15		4.030907				−40.3438			
	STR0100	0.1									
	CORR_D	0.025									
	CORR_V	0									Vertical correction
	2*CORR_D	0.05									
	CORR_H	0									Horizontal correction
	CORR_D	0.025									
	STRx203	0.20311									
	SD	0.1			−116.4139				1165.14		
	STR0010	0.01									
	DIP‡	1.22378	3	−0.861464			−0.52405	8.62207			
	… symmetric around DIP centre (see Fig. 1 ▶)										
UC4	= mirror identical to UC2 (stated as –UC2 in Fig. 1 ▶)										
UC5	= mirror identical to UC1 (stated as –UC1 in Fig. 1 ▶)										
S	…	1.302									
GS											
MC2	= mirror identical to MC1, except MC2 has CORR_V and CORR_H between SDend and OYY (stated as –MC in Fig. 1 ▶)										
GE											
LS	…	2.321									

† Double octupole strength stated in technical specification document, due to +100% tuning range.

‡ Both DIPm and DIP consist of 12 slices. Length and bend angle stated in this table are sum of slices, *k*, *B* and *B*′ are for central slice.

## Footnotes

1 Around the whole ring for some types (dipoles for example), or per achromat, *etc*.

2 Using the 2D magnetic field simulation code *FEMM* (Meeker, 2009 ▶).

3 Supporting information for this paper is available from the IUCr electronic archives (Reference: XE5010).

4 Except that the Mc2 yoke blocks have both *x* and *y* corrector pole roots between SDend and OYY.

5 Following a 3° arc for DIP and a 1.5° arc for DIPm, with larger radius through the soft end portion.

6 Note the order of precedence among reference planes, which defines the coordinate system alignment method in 3D coordinate measurement machine (CMM).

7 At one point for each magnet element, which is disconnected when dismounting the magnet block top half from the bottom half.

8 For the plastic covers on the inner side of the magnet blocks.

9 An initial test was made for each magnet element and the cases for which some response was seen were documented.

10 In the latter case, the field in the affected magnet has opposite sign to the field in the causing magnet.

11 Note that, compared with Table 6 ▶, the specification document only refers to the ‘standard’ versions of the yoke blocks.

12 Reference surfaces are described in §4.

13 150 mm for sextupoles, octupoles and correctors, 200 mm for QFm and QF, 300 mm for QFend and QDend.

14 Compared with Table 5 ▶, the call for tender only referred to the standard versions of the magnet blocks.

15 The recipe used was developed by Scanditronix AB, Uppsala, Sweden, in the 1960s.

16 The ‘evaluation coordinate system’ is the working coordinate system in which all results in the measurement report are calculated and in which all point data are presented.

17 The reason for setting *x* = 0 and *z* = 0 at the intersection with the *y* = 0 plane is that this is the best estimate of how the top and bottom yoke blocks align to each other by the guide blocks (see, for example, Fig. 11 ▶).

## Appendix A MAX IV 3 GeV storage ring lattice file

The lattice file constitutes the most basic specification for the magnet design, as it defines the specified magnet bend angles/focusing strengths, effective lengths, placement and distances between magnets. Table 10 ▶ presents the MAX IV 3 GeV storage ring lattice by listing lattice elements in consecutive order, together with corresponding field strengths at 3 GeV. The magnetic field terms should be understood as *B*_*y*_ components in an *x*, *y*, *s* curvilinear coordinate system where *s* is the position along the nominal beam trajectory and *x* and *y* are locally orthogonal to *s*, with +*y* = vertically upwards and +*x* = horizontal radially outwards in the storage ring.

As noted in §2, the DDR version of the lattice corresponded to the conclusion of iteration with *Radia* 3D magnetic design. Further updates to the magnet lattice came from iteration with magnet block mechanical design (Leemann, 2010*b* ▶), adjusting corrector and BPM placement, and from prototype magnet results (Leemann, 2011*a* ▶; Johansson *et al.*, 2011 ▶), after which the specified magnet strengths have not undergone any major adjustments. The magnet technical specification used for procurement (Anderberg *et al.*, 2011 ▶) corresponds to this version of the lattice (consequentially, currents and voltages constituting input to 3 GeV ring power supply specifications and cabling schematics correspond to this version of the lattice and DDR versions of the magnets.). A subsequent lattice update after iteration with detailed vacuum design (Leemann, 2011*b* ▶) contained minor adjustments of corrector locations (necessitating minor updates to the manufacturing drawings of the magnet blocks after the procurement had already been placed), but kept magnet strengths unchanged. This is the lattice presented in Table 10 ▶, constituting the 3 GeV storage-ring magnet lattice as it existed before start of production series magnetic field measurements.

## References

[bb1] Anderberg, B., Johansson, M., Lindgren, L.-J. & Fernandes Tavares, P. (2011). *MAX IV 3 GeV Storage Ring Magnets: Technical Specification.* MAX-Lab, Lund, Sweden.

[bb2] Eriksson, M. *et al.* (2011). *Proceedings of IPAC 2011*, San Sebastián, Spain, p. THPC058.

[bb3] Johansson, M. (2011). *MC Prototype Project: Results Summary.* Unpublished.

[bb4] Johansson, M. (2013). *MAX IV 3 GeV Ring Magnets 2D and 3D Simulations.* Unpublished.

[bb5] Johansson, M., Anderberg, B. & Lindgren, L.-J. (2011). *Proceedings of IPAC 2011*, San Sebastián, Spain, p. WEPO015.

[bb6] Leemann, S. C. (2010*a*). *MAX IV Detailed Design Report*, ch. 2.1–2.4, https://www.maxlab.lu.se/maxlab/max4/index.html.

[bb7] Leemann, S. C. (2010*b*). *Updates to the MAX IV 3 GeV Storage Ring Lattice*, MAX-Lab Internal Note 20101101. MAX-Lab, Lund, Sweden (https://www.maxlab.lu.se/node/999).

[bb8] Leemann, S. C. (2011*a*). *Updates to the MAX IV 3 GeV Storage Ring Lattice*, MAX-Lab Internal Note 20110117. MAX-Lab, Lund, Sweden (https://www.maxlab.lu.se/node/999).

[bb9] Leemann, S. C. (2011*b*). *Updates to the MAX IV 3 GeV Storage Ring Lattice*, MAX-Lab Internal Note 20111124. MAX-Lab, Lund, Sweden (https://www.maxlab.lu.se/node/999).

[bb10] Leemann, S. C., Andersson, Å., Eriksson, M., Lindgren, L.-J., Wallén, E., Bengtsson, J. & Streun, A. (2009). *Phys. Rev. ST Accel. Beams*, **12**, 120701.

[bb11] Lindgren, L.-J. & Anderberg, B. (2010). *MAX IV Detailed Design Report*, ch. 2.5, https://www.maxlab.lu.se/maxlab/max4/index.html.

[bb12] Meeker, D. C. (2009). *Finite Element Method Magnetics* (Version 4.2, 2 November 2009), http://www.femm.info/wiki/HomePage.

[bb13] Russenschuck, S. (2010). *Field Computation for Accelerator Magnets.* Weinheim: Wiley-VCH.

