# Supplementary material for: Magnet design for a low-emittance storage ring
Source: J Synchrotron Radiat. 2014 Aug 27;21(Pt 5):884–903. doi: 10.1107/S160057751401666X (PMC4181640; doi:10.1107/S160057751401666X)
Supplement: Supplementary file 1 [file s-21-00884-sup1.pdf]

Figure S1

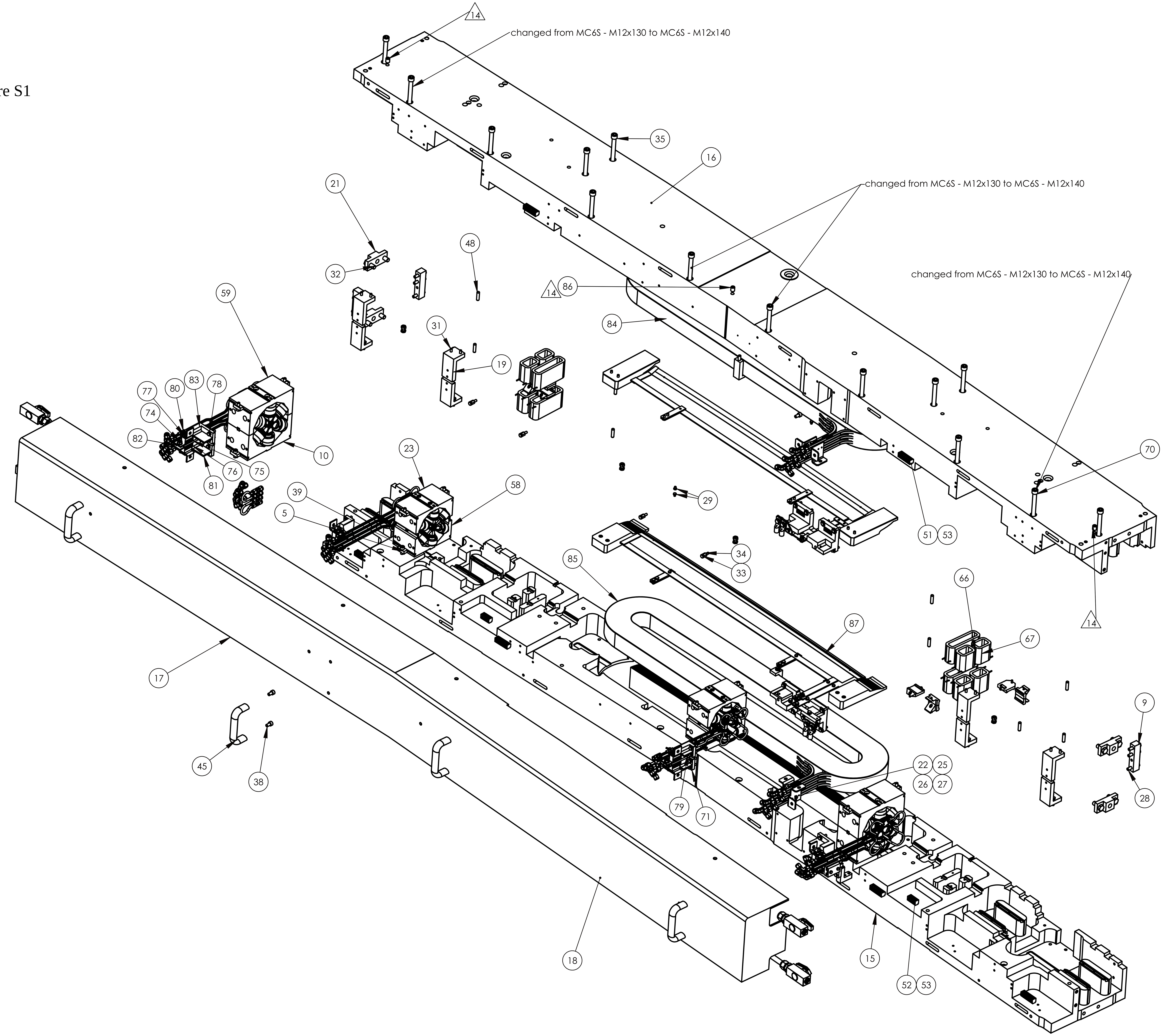

REVISIONS				
ZONE	Rev.	Description	Date	Approved
	13	2x sheets added. Item 56-63 added. Q-pole, item 23 replaced. All coils replaced. Four MC6S - M12x130 replaced with MC6S - M12x140. Jumper assemblys have been disolved and are now part of this assembly. The cable lugs with bolts, nuts and washers have been removed. Each sextupole half have been given a seperate drawing, see item 64 and 65 for the new drawing names. A sheed with water connections has beed added	2013-03-14	DL, SB
	14	3x stopscrews added. Dipole coils changed. Polarity changed for QF1 and QF2 on sheet 4 and in the trim qoil table on sheet 5. Water connections changed on sheet 6	2013-04-30	SB
	15	Thermoswitch assemblys dissolved and the terminals changeg from 2x 37 pos terminals to 1x 50 pos terminal on the top yoke and from 3x 37 pos terminals to 1x 37 pos and 1x 50 pos terminal on the bottom yoke.	2013-09-19	SB

ITEM NO.	PART NUMBER	QTY.
1	3.COAp-_DMP017, Pole top, X-corr	4
2	3.COAp-_DMP018, Pole top, Y-corr	8
3	3.DMA005, Shunt pcb subasm	1
4	3.DMA009, Cover attachment subasm	4
5	3.DMP004, Clamp, poles	16
6	3.DMP005, Coil attachment	4
7	3.DMP006, Clamp attachment	2
8	3.DMP064, Loop tube	8
9	3.U3_DMP050, Alignment tool	3
10	3.SXFI-O_DMA101, Sextupole SF0 and SFi	2
11	Water Manifold Lower U3, 201424	1
12	3.U3_DMA006, Coilbracket subasm	4
13	3.U3_DMA007, Coilbracket subasm	4
14	Water Manifold Upper U3, 201385	1
15	3.U3_DMP003, Yoke bottom, Uc3	1
16	3.U3_DMP004, Yoke top, Uc3	1
17	3.U3_DMP005, Cover, left	1
18	3.U3_DMP006, Cover, right	1
19	3.U4_DMP005, Attachment base	12
20	Q- Pole UC3 203528	16
21	3.Ux_DMP014, Attachment, lifting point	4
22	Cable-Lug-KRF-1_5-4-Nelco	18
23	3.SXD_DMA101, SD Sextupole, half	2
24	Elbow SS-6M0-9 Swagelok	44
25	Lock washer, DIN 6798 Typ A 4,3 A4	18
26	Screw M6S M4x10 A2.sldprt	2
27	Nut M6M-M4 A2	2
28	Screw M6S M8x40 A2	9
29	Screw M6S-M5x6 A2	4
30	Screw M6S-M6x12 A2	14
31	Screw M6S-M6x25 A2	56
32	Screw, M6S-M12x30H A2	8
33	Screw, M6S-M6x8 A2	4
34	Screw-DSS-M60100x12-Bufab	4
35	Screw-MC6S-M12x130 A2	10
36	Screw-MC6S-M4x12 A2	16
37	Screw-MC6S-M6x30 A2	4
38	Screw-MC6S-M8x14 A2	8
39	Screw-MC6S-M8x20 A2	16
40	Screw-MC6S-M8x25 A2	48
41	Screw-MC6S-M8x75 A2	4
42	Thermo contact, thermik L01-60	22
43	Thermo contact, thermik L01-70	22
44	Union, SS-6M0-6_6, Swagelok	16
45	WN135-120-M8-Wiberger	4
46	3.U1_DMA008, Trimcoil terminal 5-pos	1
47	3.U1_DMA008, Trimcoil terminal 3-pos	1
48	Cylindrical pin CP 8m6x30 A4	10
49	Screw M6S-M8x16 A2	4
50	Nut M6M-M8 A2	12
51	Multi terminal strip MK3/6 Weidmüller 0274220000	8
52	Multi terminal strip MK3/4 Weidmüller 0274020000	4
53	MC6S M3x16 A2	26
54	Phoenix plug in 16p1757158	3
55	Phoenix plug-in 8p1757077	1
56	Phoenix plug-in 4p1757035	1
57	Multi terminal strip MK3/8 Weidmüller 0274420000	1
58	3.SXD_DMA104, SD Sextupole, half	2
59	3.SXFI-O_DMA104, Sextupole SF0 and SFi	2
60	Coil 1 brazed UX_DMP004, Danfysik, 7103014061	2
61	Coil 4 brazed UX_DMP004, Danfysik, 7103014064	2
62	Coil 7 brazed UX_DMP004, Danfysik, 7103017270	2
63	Coil 6 brazed UX_DMP004, Danfysik, 7103017124	2
64	Coil 5 brazed UX_DMP004, Danfysik, 7103017123	2
65	Coil 8 brazed UX_DMP004, Danfysik, 7103017265	2
66	Coil COAP_DMP002, Danfysik, 7103013516	4
67	Coil COAP_DMP014, Danfysik, 7103013517	8
68	Coil 3 brazed UX_DMP004, Danfysik,7103014063	2
69	Coil 2 brazed UX_DMP004, Danfysik, 7103014062	2
70	Screw-MC6S-M12x140 A2	4
71	3.M1_DMP032, Jumper bracket	6
72	3.U3_DMP023, Jumper spacer	4
73	Washer BRB-8,4 A2	16
74	Screw M6S-M8x25 A2	8
75	3.M1_DMP018, Marking [Positive]	8
76	3.M1_DMP018, Marking [Negative]	8
77	Screw M6S-M5x12 A2	64
78	Washer BRB-6,4 A2	32
79	3.M1_DMP033, Jumper bracket	6
80	Screw M6S 4x6 A2	16
81	3.M1_DMP023, Jumper bracket	2
82	3.U3_DMP023, Jumper spacer	4
83	3.M1_DMP019, Jumper bracket	2
84	Coil upper UX_DMP012, Danfysik, 7103014117	1
85	Coil lower UX_DMP012, Danfysik, 7103014116	1
86	ISO 4029 A2 M12x16	3
87	204590	1
88	D-Sub 50p Male	1
89	Phoenix contact 2304063	1
90	Phoenix contact 2304076	2
91	DIN mounting rail, B=35 H=7,5 L=213	1
92	DIN mounting rail, B=35 H=7,5 L=261	1

Where used: 3.U3_DMA001, Uc3			External BOM				
Designed by: RL	Designed date: 2010-10-08	Material: -	Surface treatment: -			Edges broken: 0,2	
Reviewed by: -	Reviewed date: -	Approved by: -	Approved date: -	General tolerance SS-ISO 2768-1: fine	General surface finish, Ra: 3,2	Projection type	Scale: 1:5
MAX-lab			Description: Uc3, assy				
			Drawing number: 3.U3_DMA002			Revision: 15	Sheet: 1(6)

2010-10-12

This document is our property and shall not without our permission in writing be altered, copied, used for manufacturing or communicated to any other person or company.

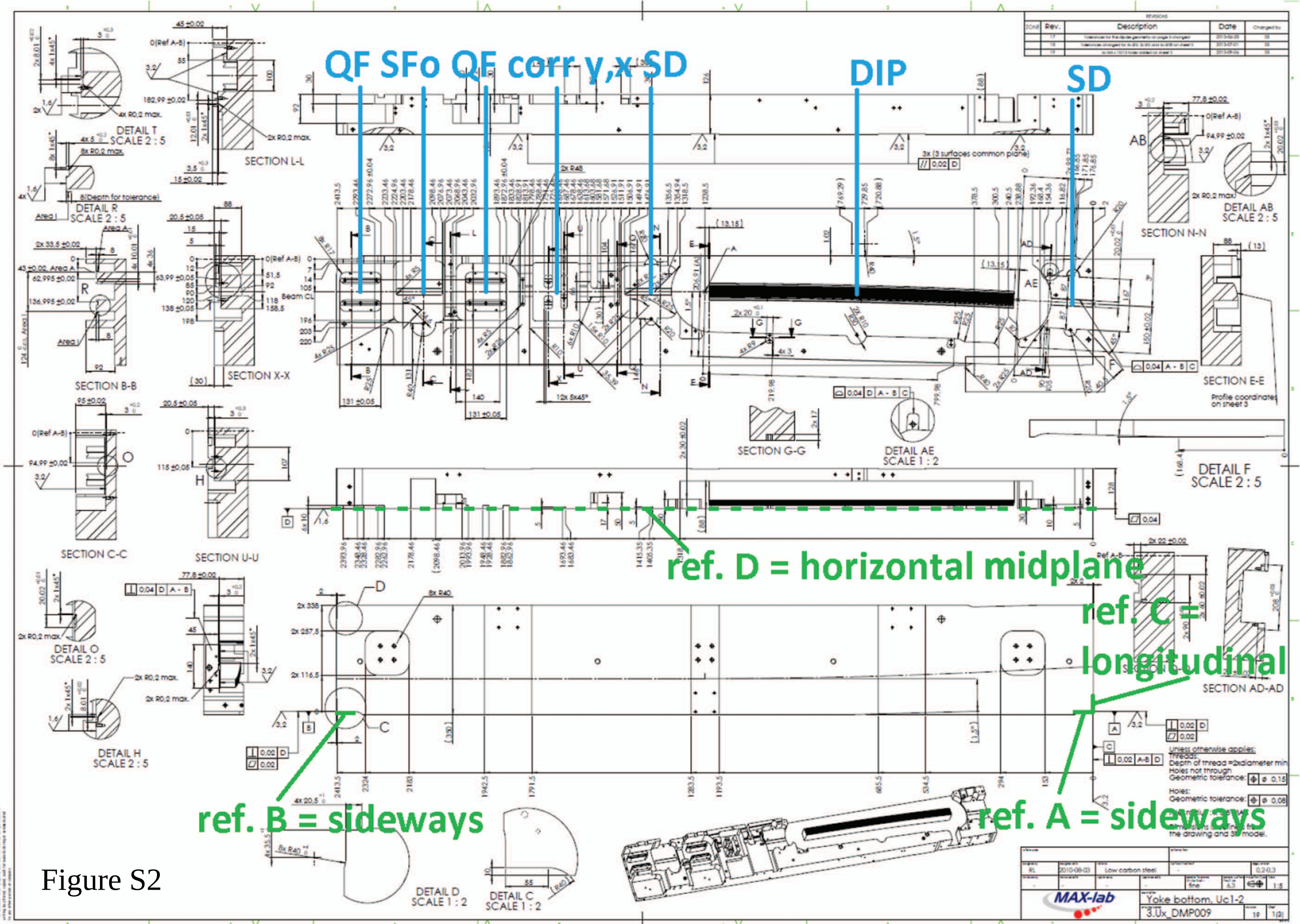
QF SFo QF corr y,x SD
DIP
SD
ref. D = horizontal midplane
ref. C = longitudinal
ref. B = sideways
ref. A = sideways
SECTION L-L
DETAIL T SCALE 2 : 5
DETAIL R SCALE 2 : 5
SECTION B-B
SECTION X-X
SECTION C-C
SECTION U-U
DETAIL O SCALE 2 : 5
DETAIL H SCALE 2 : 5
SECTION N-N
DETAIL AB SCALE 2 : 5
SECTION E-E
Profile coordinates on sheet 3
SECTION G-G
DETAIL AE SCALE 1 : 2
DETAIL F SCALE 2 : 5
SECTION AD-AD
DETAIL D SCALE 1 : 2
DETAIL C SCALE 1 : 2
Unless otherwise applies:
Low carbon steel
MAX-lab
Yoke bottom, Uc1-2
3.Ux_DMP009

Figure S2

ZONE	Rev.	Description	Date	Approved
	3	Geometric tolerance added and some dimension tolerances changed	2011-06-17	
	4	Flatness tolerance added. Dimension to holes moved. Height of ledge was 5.	2012-01-30	DL

Figure S3a

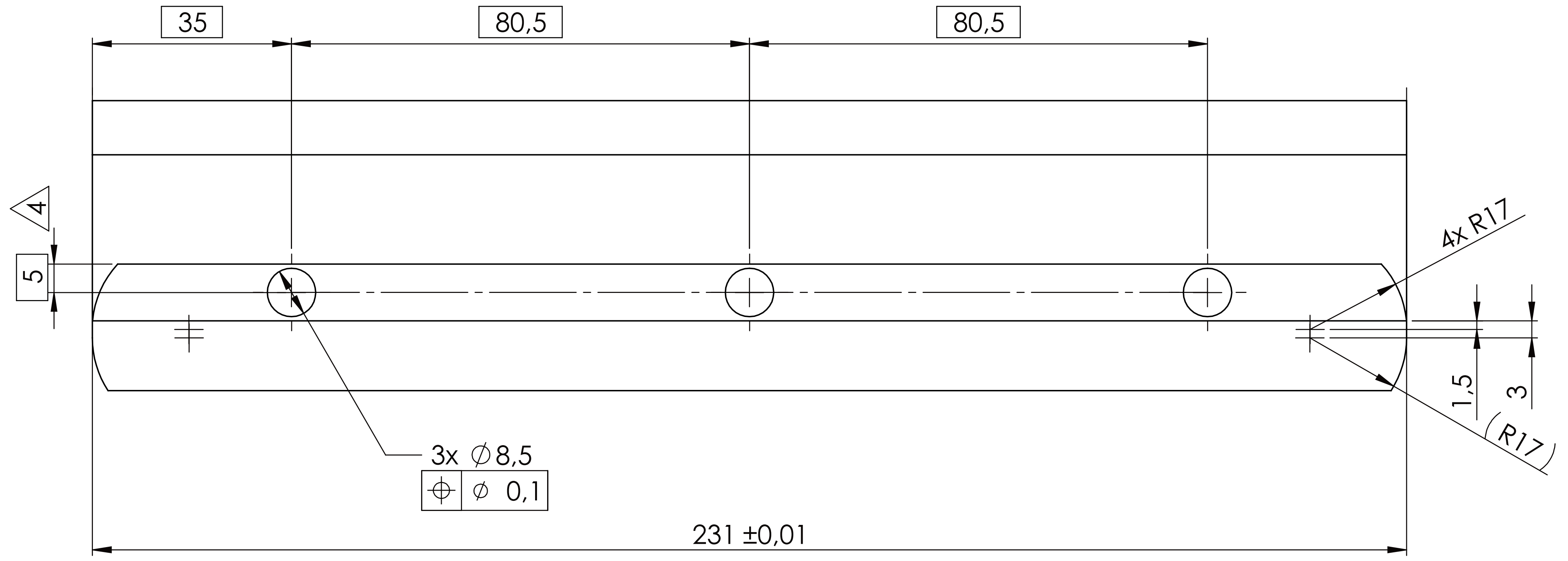

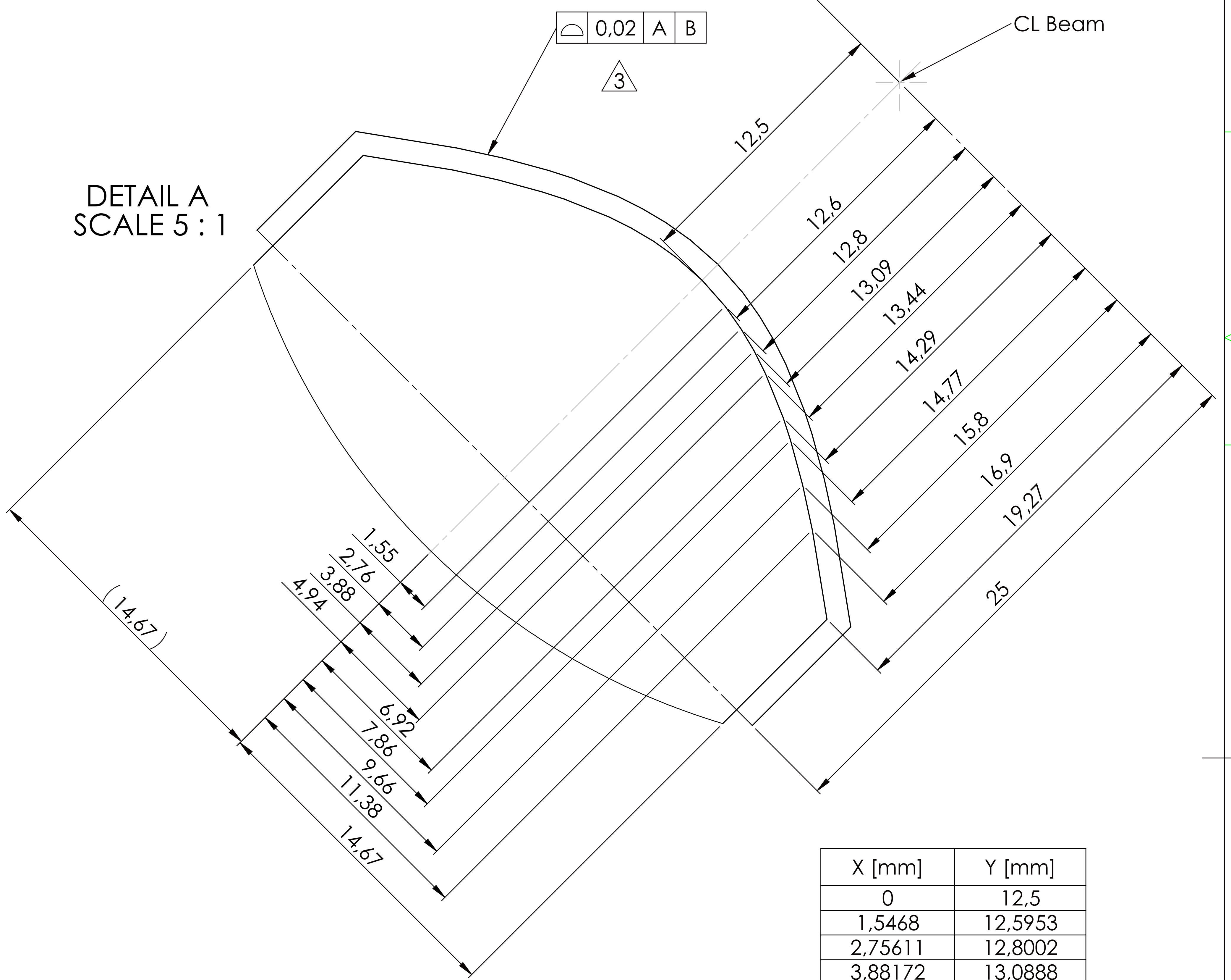

X [mm]	Y [mm]
0	12,5
1,5468	12,5953
2,75611	12,8002
3,88172	13,0888
4,94295	13,4418
6,92376	14,2894
7,86104	14,7664
9,65888	15,797
11,38	16,9043
14,6688	19,2723

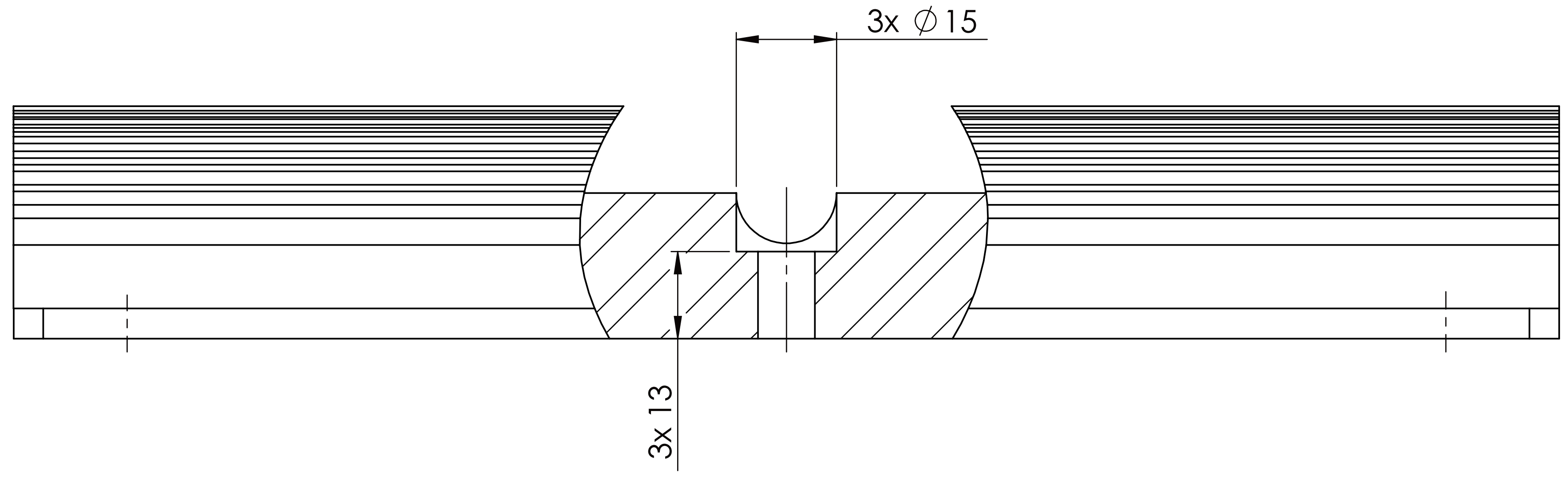

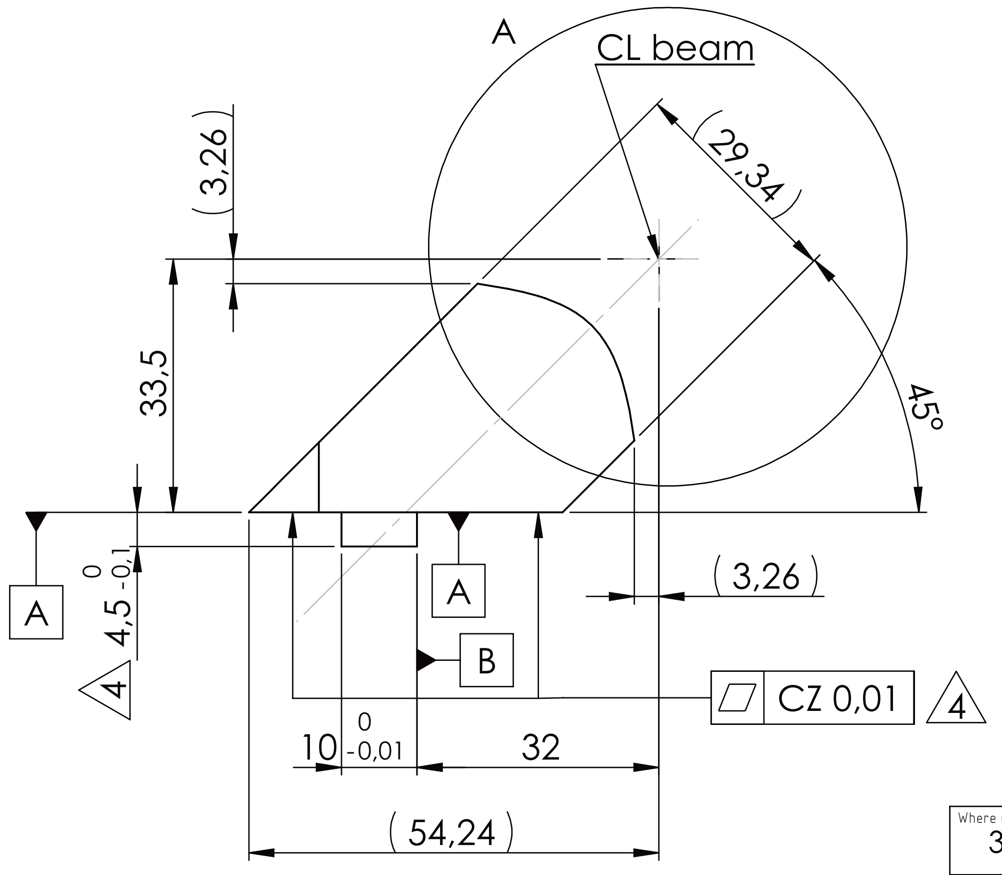

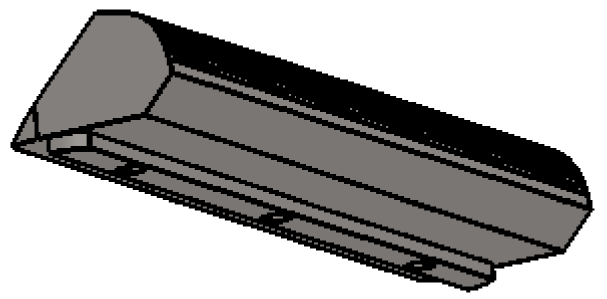

Where used	External BOM
3.M1,2_DMA002	-

Designed by	Designed date	Material	Surface treatment	Edges broken
MaBe	2010-08-17	Low carbon steel	-	0,2-0,3

Reviewed by	Reviewed date	Approved by	Approved date	General tolerance SS-ISO 2768-1	General surface finish, Ra	Projection type	Scale
-	-	-	-	fine	3,2		1:1

MAX-lab

Description		
Q-pole, Mc		
**Drawing number**	**Revision**	**Sheet**
3.Mx_DMP002	4	1(1)

This document is our property and shall not without our permission in writing be altered, copied, used for manufacturing or communicated to any other person or company.

ZONE	REV.	DESCRIPTION	DATE	APPROVED
	2	Coordinates for profile added. 6x cyl pins added.	2012-02-28	DL
	3	1x dimension removed. 2x dimension put in parentheses. Detail views changed letters.	2012-04-20	DL

REVISIONS (table above)

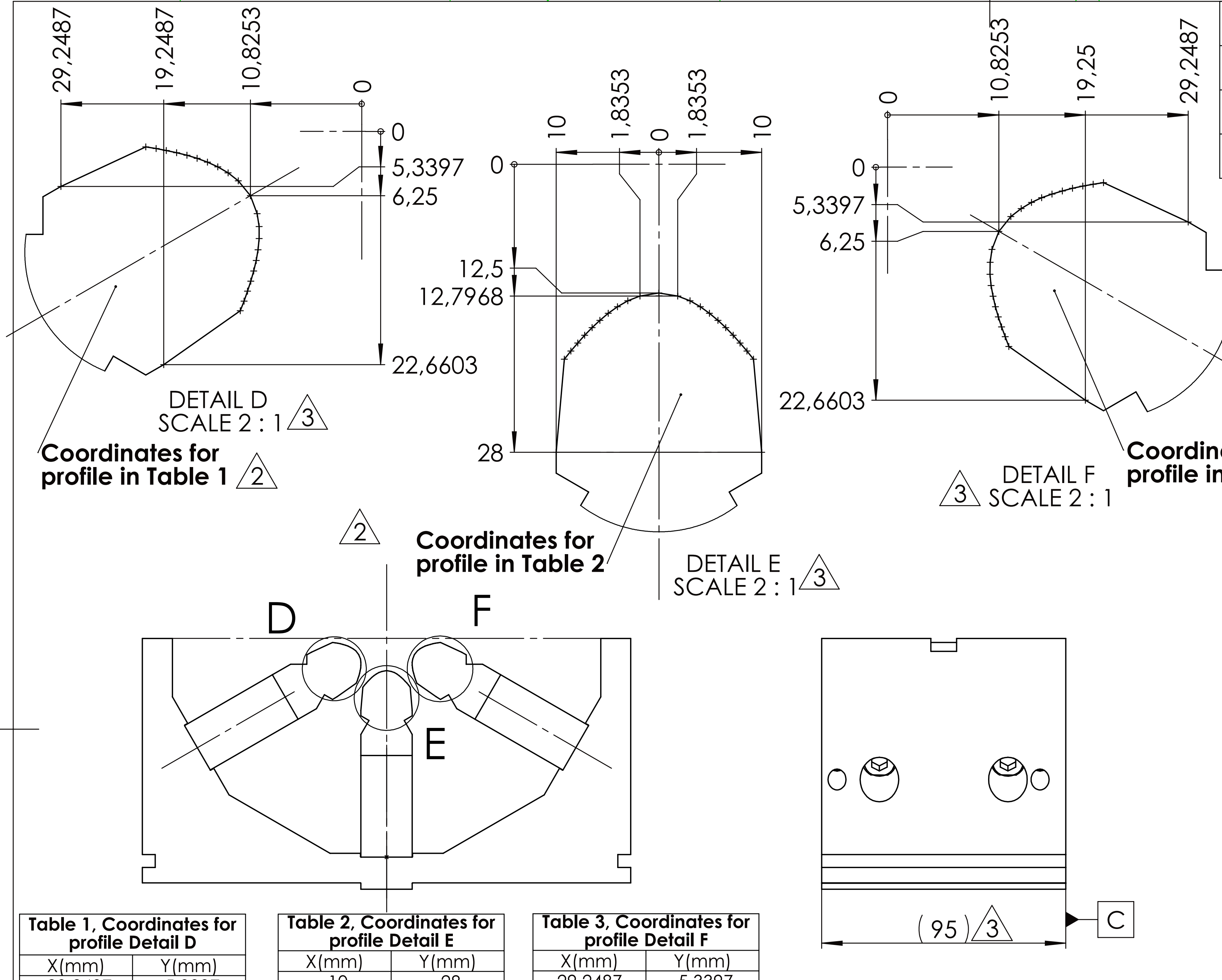

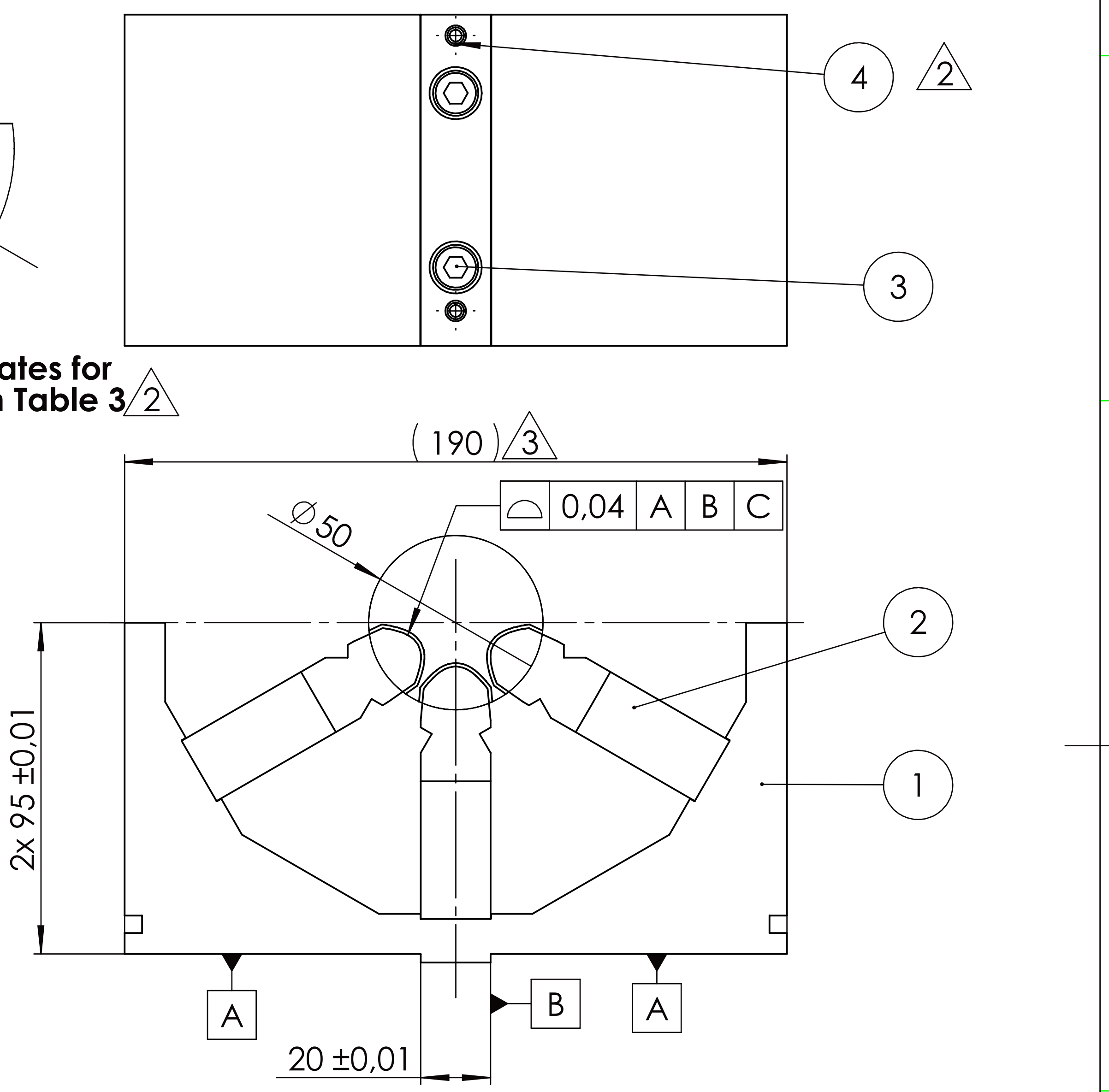

Table 1, Coordinates for profile Detail D	
X(mm)	Y(mm)
-29,2487	-5,3397
-21	-1,5101
-20	-1,666
-19	-1,8468
-18	-2,0586
-17	-2,3093
-16	-2,6102
-15	-2,977
-14	-3,4339
-13	-4,0201
-12	-4,809
-10,8253	-6,25
-10,1647	-7,9878
-9,9815	-9,2483
-9,9738	-10,4074
-10,0782	-11,5019
-10,2605	-12,5513
-10,4999	-13,5678
-10,7827	-14,5591
-11,0994	-15,5311
-11,4428	-16,4875
-11,8078	-17,4315
-19,2487	-22,6603

Table 2, Coordinates for profile Detail E	
X(mm)	Y(mm)
-10	-28
-9,1923	-18,9416
-8,5572	-18,1535
-7,9006	-17,3779
-7,2172	-16,6177
-6,5001	-15,8771
-5,7395	-15,1615
-4,9218	-14,4789
-4,0262	-13,8413
-3,0185	-13,2684
-1,8353	-12,7968
0	-12,5
1,8353	-12,7968
3,0185	-13,2684
4,0262	-13,8413
4,9218	-14,4789
5,7395	-15,1615
6,5001	-15,8771
7,2172	-16,6177
7,9006	-17,3779
8,5572	-18,1535
9,1923	-18,9416
10	-28

Table 3, Coordinates for profile Detail F	
X(mm)	Y(mm)
29,2487	-5,3397
21	-1,5101
20	-1,666
19	-1,8468
18	-2,0586
17	-2,3093
16	-2,6102
15	-2,977
14	-3,4339
13	-4,0201
12	-4,809
10,8253	-6,25
10,1647	-7,9878
9,9815	-9,2483
9,9738	-10,4074
10,0782	-11,5019
10,2605	-12,5513
10,4999	-13,5678
10,7827	-14,5591
11,0994	-15,5311
11,4428	-16,4875
11,8078	-17,4315
19,2487	-22,6603

ITEM NO.	PART NUMBER	QTY.
1	3.SXFM_DMP001, Yoke, Sextupole SFm	1
2	3.SXFM_DMP002, Pole, Sextupole SFm	3
3	Screw-MC6S-M8x20 A2	6
4	Cyl pin DIN7979 m6 6x24	6

Figure S3b

Where used				External BOM			
3.SXFM_DMA001, SFm Sextupole half							
Designed by: RL	Designed date: 2011-06-20	Material: -		Surface treatment: -		Edges broken: 0,2-0,3	
Reviewed by: -	Reviewed date: -	Approved by: -	Approved date: -	General tolerance SS-ISO 2768-1: fine	General surface finish, Ra: 3,2	Projection type	Scale: 1:2
MAX-lab			Description: Yoke half, assembly				
			Drawing number: 3.SXFM_DMA003			Revision: 3	Sheet: 1(1)

This document is our property and shall not without our permission in writing be altered, copied, used for manufacturing or communicated to any other person or company.

2010-10-12
